# Cancer Cells Hijack PRC2 to Modify Multiple Cytokine Pathways

**DOI:** 10.1371/journal.pone.0126466

**Published:** 2015-06-01

**Authors:** Mohamed Abou El Hassan, Katherine Huang, Manoja B. K. Eswara, Michael Zhao, Lan Song, Tao Yu, Yu Liu, Jeffrey C. Liu, Sean McCurdy, Anqi Ma, Joan Wither, Jian Jin, Eldad Zacksenhaus, Jeffrey L. Wrana, Rod Bremner

**Affiliations:** 1 Lunenfeld Tanenbaum Research Institute, Mt Sinai Hospital, Toronto, Ontario, Canada; 2 Toronto General Research Institute, University Health Network, Toronto, Ontario, Canada; 3 Department of Lab Medicine and Pathobiology, University of Toronto, Toronto, Ontario, Canada; 4 Department of Structural and Chemical Biology, Icahn School of Medicine, Mt Sinai Hospital, New York, New York, United States of America; 5 Toronto Western Research Institute, University Health Network, Toronto, Ontario, Canada; 6 Department of Ophthalmology and Vision Science, University of Toronto, Toronto, Ontario, Canada; H. Lee Moffitt Cancer Center & Research Institute, UNITED STATES

## Abstract

Polycomb Repressive Complex 2 (PRC2) is an epigenetic regulator induced in many cancers. It is thought to drive tumorigenesis by repressing division, stemness, and/or developmental regulators. Cancers evade immune detection, and diverse immune regulators are perturbed in different tumors. It is unclear how such cell-specific effects are coordinated. Here, we show a profound and cancer-selective role for PRC2 in repressing multiple cytokine pathways. We find that PRC2 represses hundreds of IFNγ stimulated genes (ISGs), cytokines and cytokine receptors. This target repertoire is significantly broadened in cancer vs non-cancer cells, and is distinct in different cancer types. PRC2 is therefore a higher order regulator of the immune program in cancer cells. Inhibiting PRC2 with either RNAi or EZH2 inhibitors activates cytokine/cytokine receptor promoters marked with bivalent H3K27me3/H3K4me3 chromatin, and augments responsiveness to diverse immune signals. PRC2 inhibition rescues immune gene induction even in the absence of SWI/SNF, a tumor suppressor defective in ~20% of human cancers. This novel PRC2 function in tumor cells could profoundly impact the mechanism of action and efficacy of EZH2 inhibitors in cancer treatment.

## Introduction

Cancer cells employ various strategies to evade the immune system, including regulation of cytokines or other secreted factors/receptors that control the immune response [1]. Notably, the type, location, and degree of immune infiltrate in a tumor (the “Immunoscore”) predict outcome better than a multitude of traditionally utilized tumor-centric pathology parameters, such as tumor grade [2]. The factors that control immune surveillance vary contextually and the details are complex, because agents that promote immune clearance in one situation promote immune suppression in another (reviewed in [3]). An appealing notion is that tumors might utilize a common mechanism to manipulate expression of immune regulators, and that each tumor tailors this strategy to suit their individual environment. Disrupting such a higher level regulatory network could provide a general strategy to increase immune detection and clearance of many cancers. However, the mechanisms that control the myriad of immune genes that influence surveillance are not well understood.

Polycomb Repressive Complex 2 (PRC2) is the epigenetic regulator that deposits repressive histone H3 lysine 27 (H3K27me3) marks on chromatin. Two of its major components include the catalytic subunit EZH2, and SUZ12, the scaffold protein required for complex stability [4]. PRC2 is part of the Polycomb family of regulators that counter the positive transcriptional effects of Trithorax family members during development, such as the SWI/SNF chromatin remodeling complex [5]. PRC2, which is often overexpressed in cancer, is thought to promote tumorigenesis through regulation of the cell cycle, DNA replication, survival, senescence and/or stemness [5–7]. Whether and to what extent PRC2 might influence the immune program in tumors is unclear.

Previously, we and others showed that BRG1, the ATPase engine that drives SWI/SNF, is required for responsiveness of IFNγ stimulated genes (ISGs) [8–11]. At the *CIITA* locus, we found that BRG1 coordinates the action of many distal enhancers. However, despite being essential at the endogenous locus and a large 190 kb reporter, BRG1 is dispensable for IFNγ induction of short *CIITA* reporters, leading to the notion that it may temper the effects of a remote repressor. In recent work, parallel to the current study, we showed that PRC2 and H3K27me3 decorate the *CIITA* locus, both at the promoter and between remote enhancers [12]. Removing PRC2 alleviated the requirement for BRG1, and poised a remote -50 kb enhancer, exactly as seen with BRG1. We wondered if this antagonism between BRG1 and PRC2 might extend to other IFNγ targets. IFNγ plays a vital role in immune surveillance [13–20]. 20% of human cancers lack functional SWI/SNF [21], and this defect or up-regulation of PRC2 could provide a general mechanism for immune escape in cancer. Moreover, targeting of PRC2 to distinct loci could help sculpt the specific immune program required in different cancers.

Here, we show that PRC2 has a broad role in repressing ISGs, and unexpectedly that it also has a dramatic and cancer-selective role in regulating many other cytokine pathways. Inhibiting PRC2 with RNAi or small molecule EZH2 inhibitors reactivated ISG responsiveness, even in SWI/SNF-deficient cancer cells. Moreover, extensive RNAseq analysis revealed that disrupting PRC2 activates multiple cytokine and cytokine receptor pathways. This function was considerably expanded in cancer *vs*. non-cancer cells, and could be blocked through pharmaceutical means. PRC2 is thus a higher order regulator of immune pathways in cancer, targeting cytokines, their receptors, and downstream targets, and it tailors this program according to cancer type. Cytokines have pleiotropic effects on tumorigenesis [3,22], and could have a major influence on the efficacy of EZH2 inhibitors in human cancer.

## Material and Methods

### Cell Culture and Adenoviruses

Cells were grown as described [9] and treated with human IFNγ (Invitrogen) at a concentration of 0.1 mg/ml. SW-13 cells were transduced with adenovirus expressing GFP (Ad-GFP) or GFP-BRG1 fusion protein (Ad-BRG1) described [9]. AdBRG1 virus was titrated so that BRG1 expression matched that in HeLa cells [9].

### Small Interfering RNA (siRNA)

siSUZ12, siEZH2 and siCtrl were obtained from Qiagen, and siBRM from Thermo Scientific. SW-13 cells at 2x10^6^ per 10-cm dish were transfected with 50 nM siCtrl, siSUZ12 or siEZH2 alone or with 75 nM siBRM for 3–4 days using DharmaFECT-1 (Thermo Scientific). Cells were sub-cultured at 2x10^6^ per 10 cm dish and subjected to a second transfection cycle, ensuring better H3K27me3 depletion. Subsequently, cells were treated with 0.1 mg/ml human IFNγ for 6 hours.

### Westerns

Westerns were performed as described [23]. For antibodies see S4 Table.

### RT-PCR and Expression Arrays

RNA was reverse-transcribed and analyzed by quantitative PCR. Values were normalized to β-actin [9]. Primers are in S4 Table. For expression arrays, RNA quality was checked using Bioanalyzer (Agilent Inc.), converted to cDNA, followed by 2^nd^ strand synthesis and cRNA preparation using Ambion kit (Applied Biosystems). cRNA was column-purified quality checked using Agilent Bioanalyzer. 1.5 μg was hybridized to Illumina whole-genome expression arrays. AdGFP/BRG1 and siCtrl/siSUZ12 samples were hybridized to Human-WG6 Expression BeadChips and Human-HT-12 Expression BeadChips, respectively (Illumina, Inc.). Differential expression analysis was performed using average normalization by BeadStudio software (Illumina Inc). Three biological replicates were included for each treatment group.

### ChIP qPCR, ChIP on chip, and ChIP-seq

ChIP-qPCR DNA was performed as described [10,24]. Antibodies and primers are in S3 & S4, respectively. Antibodies were previously validated [25] and we further verified H3K27me3 and H3K79me3 antibodies using dot blots. For Chip-chip, immunoprecipitated DNA was amplified, labeled, and hybridized to Human 1M promoter tiling arrays (Agilent). TileMap was used to define regions with significantly enriched H3K27me3 [26], and intensities were normalized to internal standards. Peaks were ranked according to their test statistics value (maximal TileMap-MA statistic: maxM/P [26]). Methods to set a maxM/P cutoff are described in S1 Text (Supplementary Methods). For details on the analysis of ChIP-seq data, see S1 Text (Supplementary Methods).

### RNA-Sequencing

RNA quality was tested using BioAnalyzer (Agilent). Oligo-dT beads were used to isolate poly(A)^+^ mRNA, and after random fragmentation ~ 300bp RNA fragments were used to prepare multiplexed cDNA libraries using Illumina TruSeq RNA Sample Preparation Kit. High-throughput sequencing of libraries was performed using Illumina HiSeq 2000 at LTRI. Sequencing reads of each sample in fastq format were assessed with FASTQC, and per base sequence quality and per sequence GC content indicated high quality data. Data were mapped onto the human genome (hg19) using TopHat 1.4.1 that allows for up to two mismatches [27]. Non-unique reads were filtered out. The reads count (or assembly) for each gene was calculated using a custom R-based pipeline ((http://www.r-project.org). For further details on how data quality and differentially expressed genes were assessed see S1 Text (Supplementary Methods and Results).

### Gene Set Enrichment Analysis

We extracted genes with log_2_Fold change > 0 and differential probabilities > 0.5 and ranked the SUZ12 suppressed genes by decreasing differential probability values. We used the GSEA Preranked function to analyze the enrichment of the ranked genes on the C2 gene sets exported from KEGG pathway version 3.1 with the genes in the gene sets identified by HUGO gene symbol. We selected enriched pathways with high Enrichment Score (ES) by a cutoff of nominal P value (NOM p-val) < 0.01 and FDR q-val < 0.05. For the “cytokine-cytokine receptor interaction” (CCRI) pathway, we further applied GSEA Leading-Edge analysis to extract pathway members that explain the enrichment. We clustered the genes with their DE probability across the six cancer cell lines to common and differently affected genes in different cancer cells within the same pathway. We used the KEGG pathway map of the CCRI pathway (http://www.genome.jp/kegg/pathway.html, map 04060) to generate schematics. Clustering analysis and visualization were conducted with MeV (http://www.tm4.org/mev.html).

### Cytokine Detection

For Suz12 knockdown, A549 cells were treated for two-cycles as per above. For drug inhibition of Ezh2, cells were treated for two four day cycles with 2 uM GSK343 or UNC1999. At the end of each treatment, cells were seeded @ 3x10 ^4^ / well in 96 well dishes. Cells were allowed to settle for 4 h and then the indicated concentrations of the various stimuli (LPS, IFNγ, IL1β and TNFα) were added. Culture supernatants were collected after 24 h and frozen until use. ELISA was performed in duplicate using IL6-, IL8- and CXCL10-ELISA Max Deluxe kits from Biolegend. Multiplex analysis of cytokines in culture supernatants was performed using the Human Primary Cytokine Array/Chemokine Array 41-Plex assay from Eve Technologies Corporation, Canada. Three independent experiments were performed. Western blot analysis was performed to ensure Suz12 knock down and down regulation of H3K27me3 after each experiment.

## Results

### Genome-Wide Antagonism between BRG1 and PRC2 at IFNγ Targets

**Fig 1:**
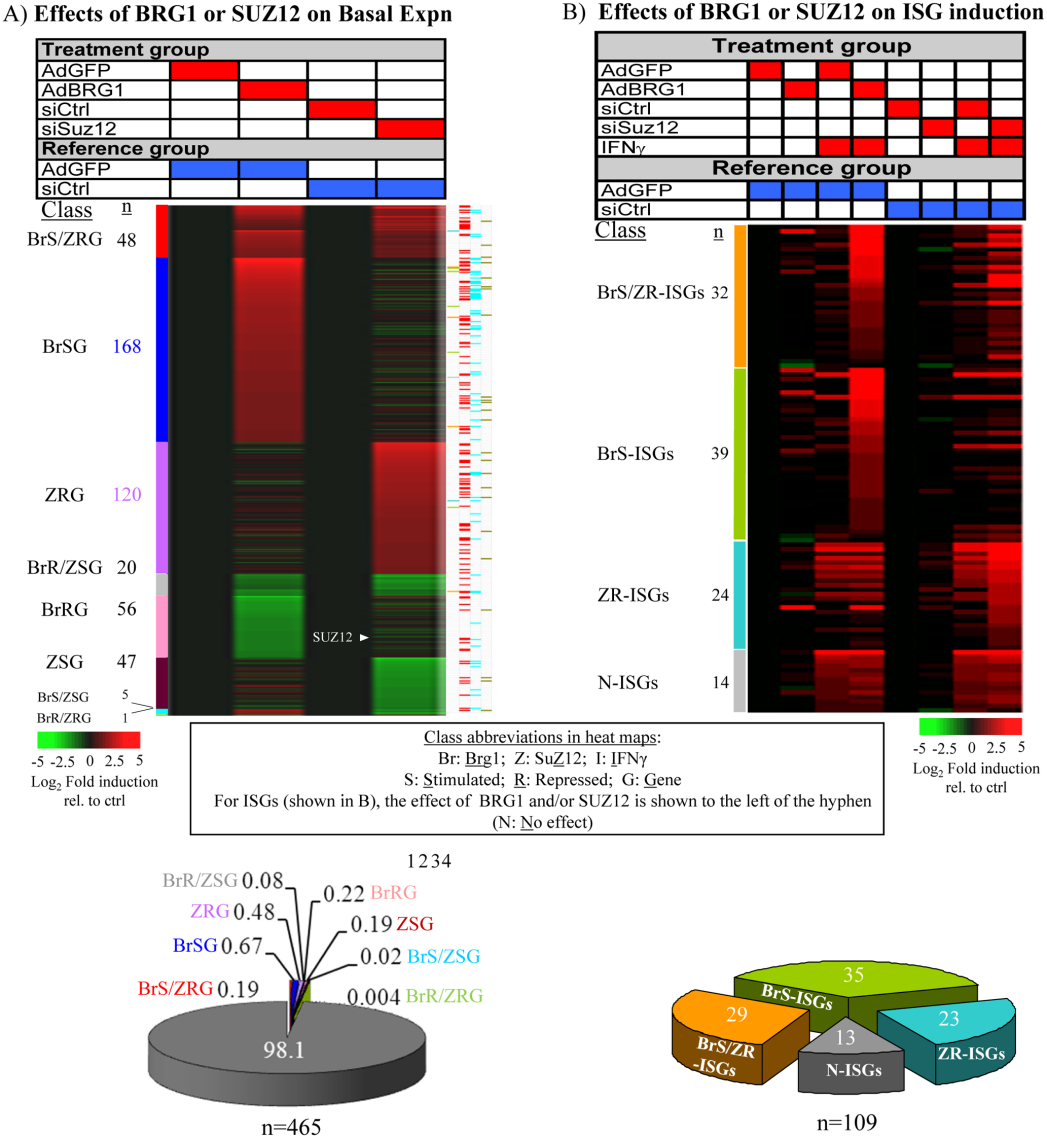
BRG1 and PRC2 regulate most ISGs. Microarrays were performed with RNA from SW-13 cells to assess the effect of BRG1-reconstitution or SUZ12 knockdown on basal expression of all genes (**A**, 465 affected genes) or IFNγ responsiveness (**B**, 109 ISGs). Treatments are indicated in red and blue above each heatmap, gene classes are indicated by colored bars to the left of each heatmap, and the pie graphs summarize the % of genes in each class (In (A) grey = unaffected genes). An additional chart to the right of the heatmap in (A) classifies genes based on: 1. IFNγ responsiveness (ISGs = 12), or GO terms: 2. Development (n = 118); 3. Cell signalling (n = 64); 4. Cell migration (n = 24). Gene Class Abbreviations: Expn: Expression; Br: Brg1, Z: Suz12; S: Stimulated; R: Repressed; I: Interferon-γ; G: Gene.

SWI/SNF regulates ISGs [8–11,28–30], but the functional relationship with PRC2 is unknown. To compare targets genome-wide, BRG1-deficient SW-13 cells were transduced with adenoviral vectors expressing BRG1 (Ad-BRG1) or GFP (Ad-GFP), or transfected with siCtrl or siSUZ12, left untreated or IFNγ-stimulated for 6 hrs, and microarrays used to assess mRNA levels. SUZ12 is a core subunit of PRC2 and its loss results in degradation of the enzymatic subunit, EZH2 [4]. We focused on genes at which AdBRG1 or siSUZ12 induced expression ≥ 2-fold (for an overall data summary, see pie charts in Fig 1). A detailed discussion of the data and gene classes is provided in S1 Text (see the first section of Supplementary Results); the latter provides extensive details on the degree to which BRG1 and PRC2 regulate basal and IFNγ induced gene expression. Strikingly, BRG1 reconstitution or siSUZ12 treatment enhanced induction of 87% (95/109) of ISGs, but affected basal expression of only ~2% of all genes (Fig 1). Moreover, of the 2% of all genes whose basal expression was affected, 14% (48/342) were co-regulated by SWI/SNF and PRC2, whereas 34% (32/95) of ISGs were co-regulated. The effect of SWI/SNF and PRC2 on co-regulated genes was antagonistic. Real time PCR analysis of 52 ISGs confirmed the predominant BRG1-dependency in this gene class (Fig A in S1 File), and siSUZ12 rescued responsiveness at 5/7 BRG1-dependent ISGs, while BRG1-independent ISGs or control genes were unaffected (Fig B, Panel B in S1 File). A second SUZ12 siRNA also rescued BRG1-dependent ISGs (Fig C in S1 File). PRC2 depletion marginally induced the BRG1-related protein BRM (Fig B, Panel A in S1 File). To assess whether this slight induction might explain induction of some ISGs we knocked down BRM. This additional step had no or only a slight effect on rescue of BRG1-dependent ISGs by siSUZ12 (Figs B, D in S1 File). Rescue of ISG-responsiveness was also not due to induction of endogenous IFNs, because RNAi treatment had no effect on STAT1 phosphorylation or IRF1 protein levels, either in SW13 cells [12], or in multiple other cell lines (see below).

If PRC2 directly regulates ISGs, these targets should exhibit H3K27me3. First, we addressed this issue using ChIP-qPCR, establishing cutoffs for subsequent genome-wide analysis. We studied 30 promoters: 10 ISGs rescued by siSUZ12 or BRG1, 6 ISGs rescued by siSUZ12 only, 6 ISGs rescued by BRG1 only, 3 ISGs unaffected by siSUZ12 or BRG1, and 5 positive controls for H3K27me3. IRF1, unaffected by either PRC2 or BRG1, lacked H3K27me3 and acted as baseline (Fig E in S1 File). H3K27me3 was detected at 15/16 of the siSUZ12 regulated ISG promoters, but only 4/9 of the ISGs unaffected by siSUZ12 (Fig E, Panel A in S1 File) (p < 0.05, Fisher exact test), and H3K27me3 was significantly higher at the former (Fig E, Panel B in S1 File).

**Fig 2:**
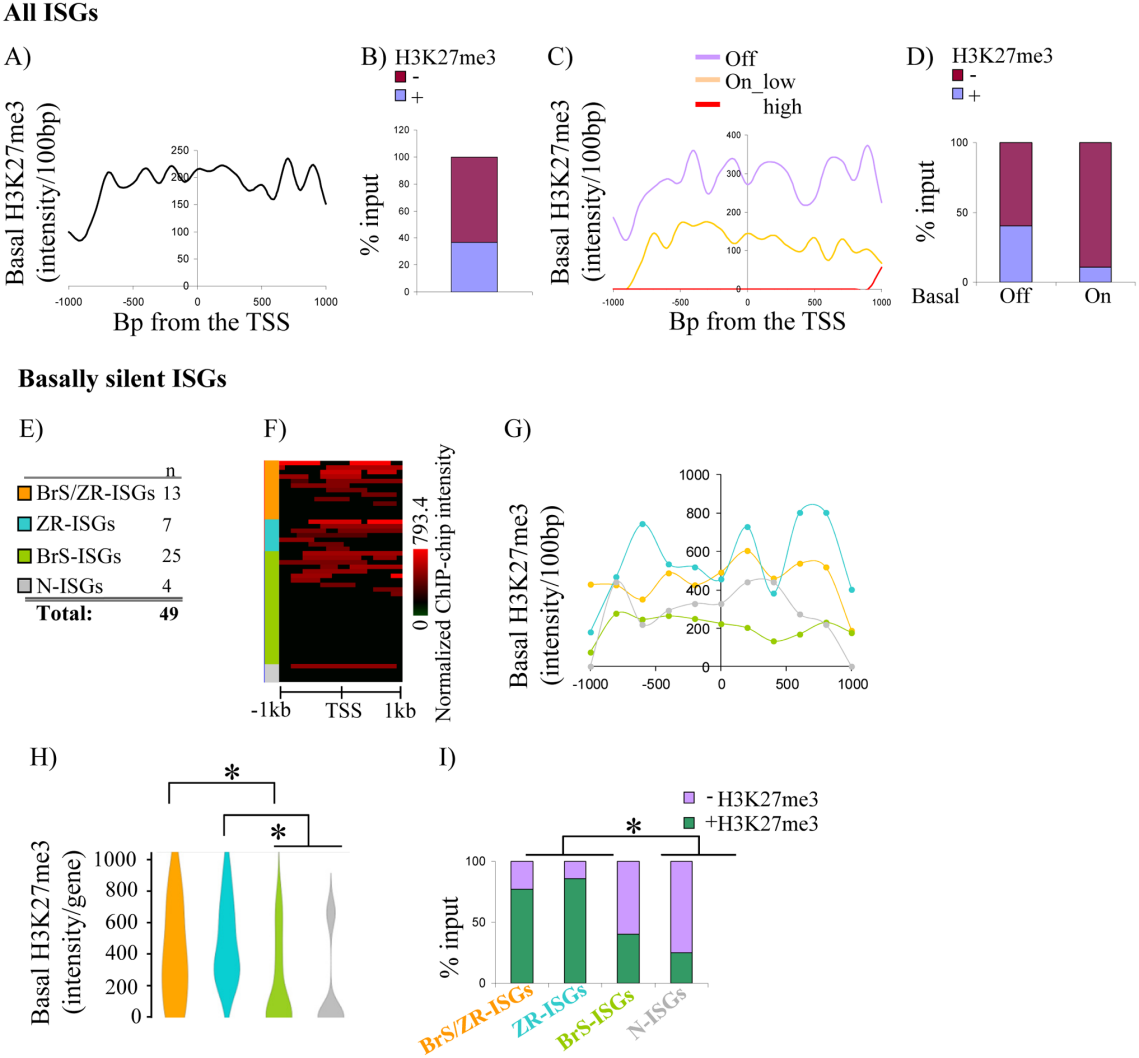
PRC2 epigenetic signature is common at ISG promoters. Genome-wide ChIP-chip data was used to assess H3K27me3 levels at ISG promoters. **(A)** ChIP-chip signal intensity per 100 bp bins within +/-5 kb of the TSS of all 109 ISGs defined in Fig 1B. **(B)** Percentage of H3K27me3 positive and negative ISG promoters. **(C)** ChIP-chip signal intensity as in (A) but grouped according to ISG basal expression. **(D)** Histogram of the percentage of H3K27me3 positive and negative ISGs in relation to their basal gene expression. **(E)** Color code of basally silent ISGs analyzed in (F)-(H). **(F)** Heatmap shows basal H3K27me3 ChIP-chip signal within +/-1 kb of the TSS of the indicated basally silent ISG classes. **(G)** ChIP-chip signal intensity per 100 bp bins within +/- 1kb of the TSS of basally silent ISGs. **(H)** Violin plot shows the level of the average ChIP-chip signal. Asterisks indicate significant difference (P < 0.05, Mann Whitney test) between the indicated groups. **(I)** Histogram shows the percentage of H3K27me3 positive promoters in each indicated ISG class. *: significantly higher % of H3K27me3 positive ISGs between the indicated groups (P < 0.05, Fisher exact test). Gene Class Abbreviations: Br: Brg1, Z: Suz12; S: Stimulated; R: Repressed; I: Interferon-γ; G: Gene.

Genome-wide ChIP-chip revealed ~1/5^th^ of promoters had some H3K27me3 (at least one 100 bp bin 1.5x above control). The average H3K27me3 intensity peaked +/- 1 kb around the TSS of all genes (Fig F Panels A, B in S1 File), and H3K27me3 levels anti-correlated with basal expression (Fig F, Panels C, D in S1 File), consistent with other cell types (reviewed in [6]). Focusing on genes at which BRG1 and/or siSUZ12 stimulated expression, those with the highest H3K27me3 levels were repressed by PRC2 (BrS/ZRG & ZRG; Fig G, Panels A-E in S1 File). The average level of H3K27me3 at ISGs was similar to that seen at all silent genes (c.f. Fig 2A and 2F, Panel A in S1 File). ~40% of ISGs had some H3K27me3 (Fig 2B), which is ~2x more than all genes (c.f. Fig F, Panel B in S1 File). As at all genes, the level and frequency of H3K27me3 correlated with basal expression (Fig 2C and 2D). Moreover, at basally silent ISGs, the level and frequency of H3K27me3 was significantly higher at loci where siSUZ12 enhanced IFNγ responsiveness (Fig 2E–2I, ZR-ISGs, BrS/ZR-ISGs).

Together, the above expression and chromatin binding data expose an extensive, antagonistic role for SWI/SNF and PRC2 in IFNγ responsiveness.

### PRC2 Represses Multiple Immune Pathways in Cancer Cells

To assess the general relevance of our findings, we performed RNAseq analysis after PRC2 depletion in 8 cancer and 3 non-cancer-derived cell lines (Cancer: A549, lung adenocarcinoma; Panc.04.03 & AsPC1, pancreatic adenocarcinomas; PC3, prostate cancer; MCF-7 & MDA-MB-231, breast cancers, HeLa, cervical cancer; and SW-13, adrenocortical cancer; Non-cancer: 184-A1, breast; BPH-1 prostate, and Beas-2B lung). Westerns revealed that SUZ12, EZH2 and/or H3K27me3 levels were typically elevated in cancer lines compared to non-cancer lines (Fig H in S1 File). BRG1/BRM levels varied between cancer and non-cancer lines with the lowest in A549, AsPC1, Panc.04.03 and 184A1 (Fig H in S1 File). EZH2 and SUZ12 levels correlated with each other but not with bulk H3K27me3, and PRC2 and BRG1 or BRM levels were uncorrelated (Fig H, Panel B in S1 File). These variable findings underline the importance of functional studies to deduce the effect of PRC2 on gene expression because simply assessing levels of the complex or the mark reveals little about its role in cancer or non-cancer cells.

Cells were treated with siCtrl or siSUZ12 and left untreated or exposed to physiologically relevant concentrations of IFNγ (0.2 ng/ml), comparable to that secreted by NK cells exposed to tumor cells [31,32]. SUZ12-depletion reduced H3K27me3 in all cell types, and increased H3K27ac in some (HeLa, MDA-MB-231, PC3), but did not affect SWI/SNF subunits (Fig I in S1 File). Next RNA-seq assays were run to assess the effect of SUZ12 knockdown on basal or IFNγ-induced gene expression (44 assays, 4 conditions x 11 lines), and the results were validated using principle component analysis and correlation analysis. The effect of the knocking down SUZ12 on gene expression was also confirmed with a backup siRNA against SUZ12. For a full discussion please see the section entitled “Validation of RNAseq data” in S1 Text (Supplementary Results), which refers to the validation data in Figs J–M in S1 File, and S2 Table.

**Fig 3:**
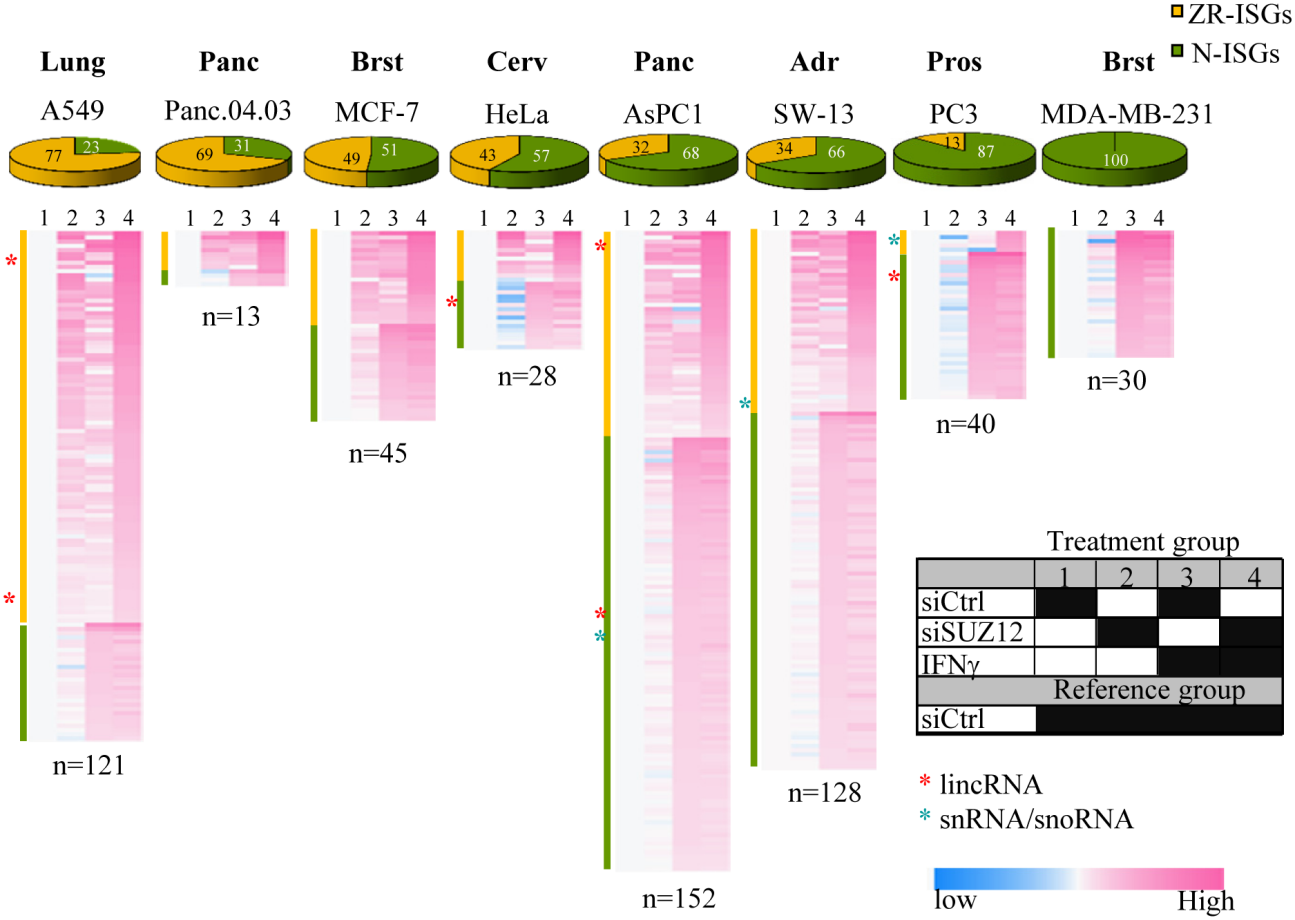
PRC2 represses ISGs in different types of cancers. Heatmaps show the effect of the four treatments (columns 1–4, key in table to right) on ISG expression in a panel of lung, pancreas, breast, cervical, adrenocortical and prostate cancer cell lines. Cells were transfected with siCtrl or siSUZ12 and left untreated or stimulated with IFNγ for 6 hours. ISGs are sorted into SUZ12-repressed ISGs (Zr-ISGs, yellow) and ISGs which are not regulated by PRC2 (N-ISGs, green). The percentage of Zr-ISGs and N-ISGs are shown in pie diagrams above each heatmap, and the total number of ISGs per line is indicated below each heatmap.

Consistent with a broad role for PRC2 at ISGs, SUZ12-Repressed ISGs (ZR-ISGs) were observed across multiple cell lines (Fig 3, Fig N in S1 File, S2 Table). Among the two most abundant ISG categories (ZR-ISGs and ISGs not affected by SUZ12 (N-ISGs)), there were cell-specific differences in absolute numbers of ZR-ISGs (range 13–152, mean 70, median 43), but siSUZ12 enhanced induction of between 13% and 77% (average 45%, median 43%) in 7/8 cell lines (Fig 3). These values are conservative, as there were other more complex lower abundance gene classes where SUZ12 affected ISG expression (S1 Table). Most N-ISGs (68%) and ZR-ISGs (72%) were specific to each cell type (Fig O in S1 File), indicating that, in addition to a core set, each line induces a distinct ISG cocktail. This is consistent with the context-specific nature of immune surveillance [3], and with cell-specific chromatin binding by PRC2 [33]; indeed of the genes repressed by SUZ12 alone (ZRG-N), 77% were unique to one cell line (Fig O in S1 File). Also, the fraction of ZR-ISGs was lower in non-cancer versus cancer lines, with the three non-cancer lines ranked 7^th^, 8^th^, and 11^th^ among all 11 lines assessed (S1 Table).

**Fig 4:**
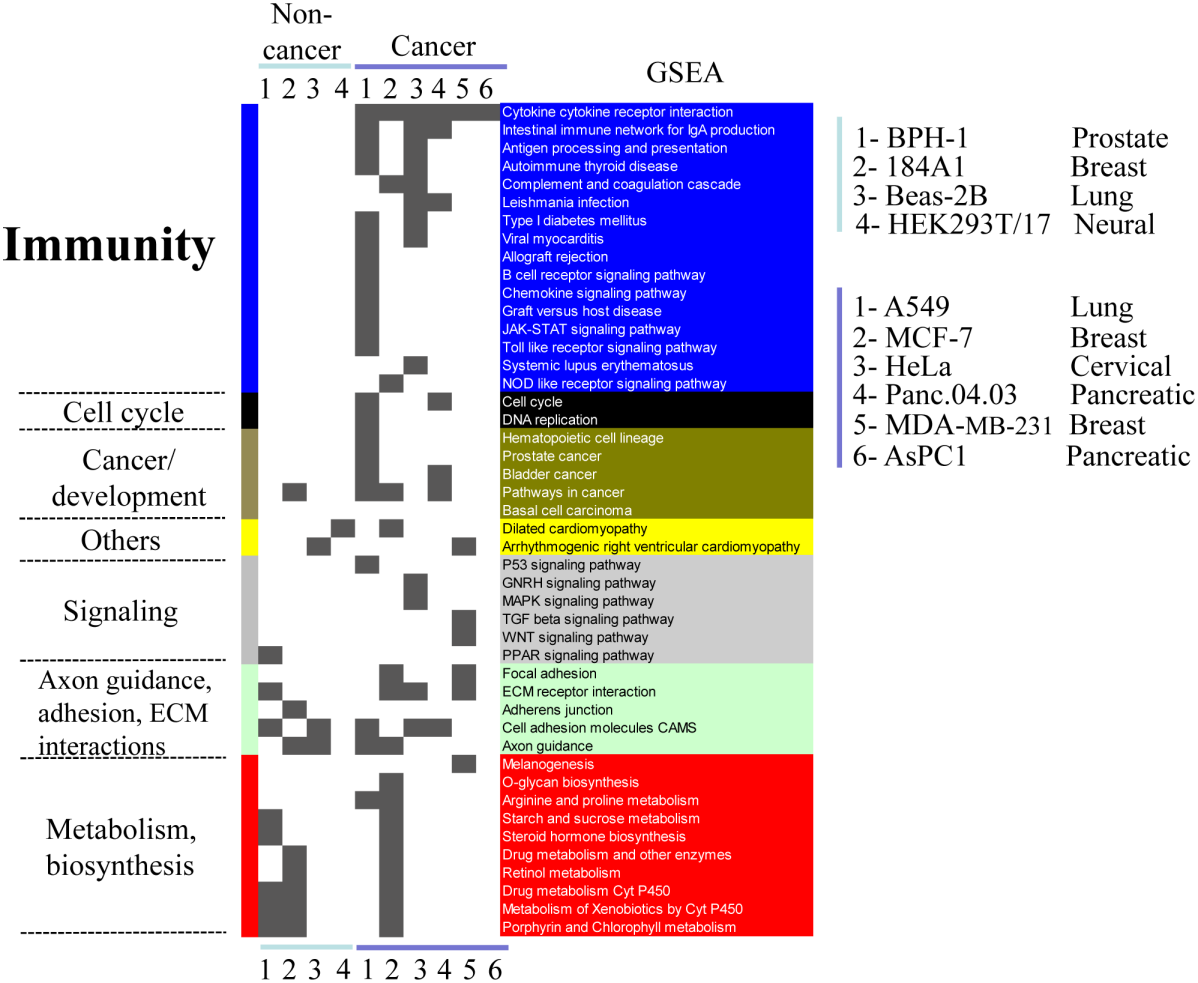
Cancer cells hijack PRC2 to regulated immune pathways. Summary of GSEA analysis of genes differentially induced by siSUZ12 relative to siCtrl in 6 cancer and 4 non-cancer derived cell lines. Cell lines included in the heatmap are indicated to the right; a significant signal is indicated in grey. Similar biological pathways are grouped, and highlighted with a distinct color to the right, and their general class name is indicated to the left.

**Fig 5:**
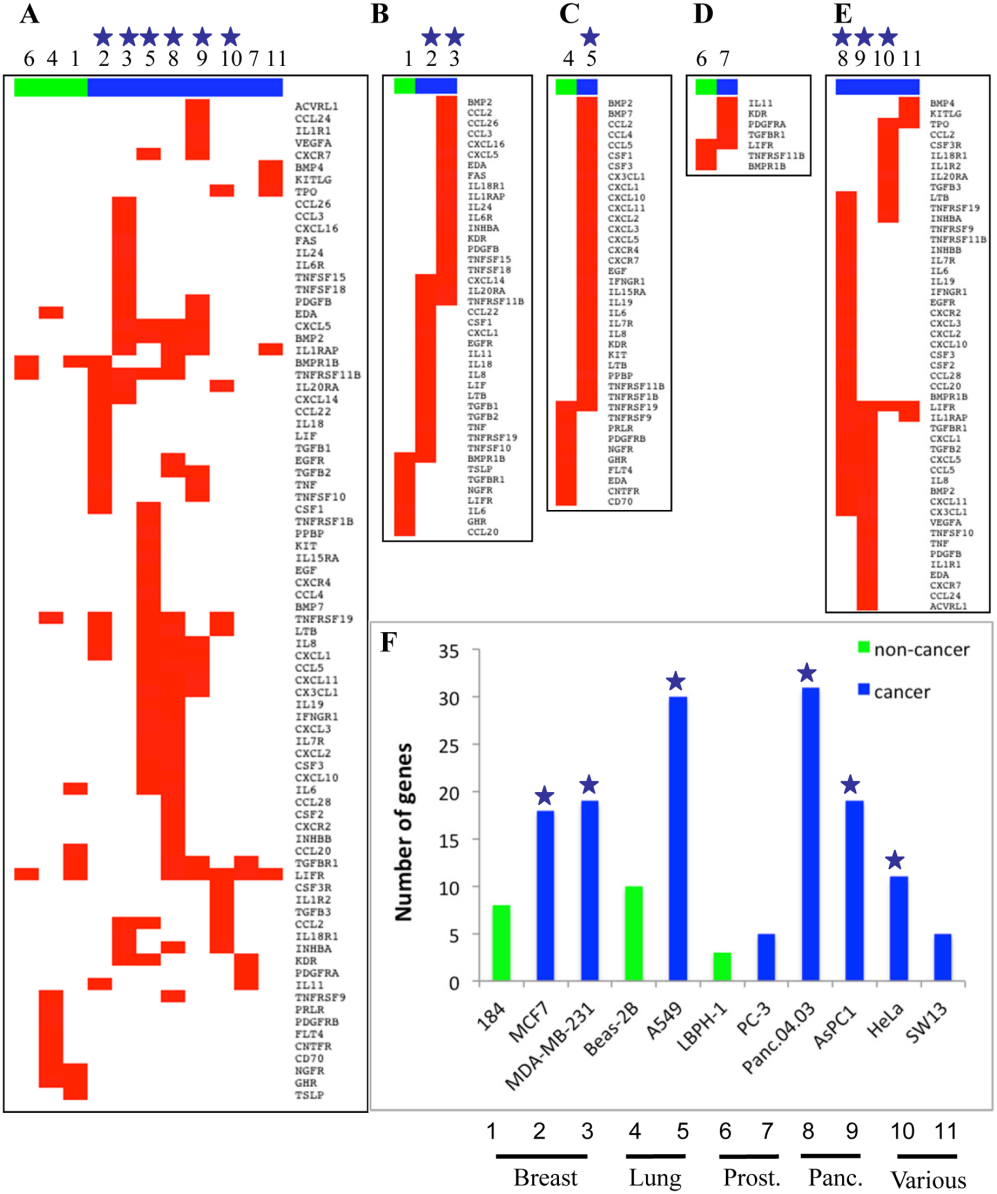
Distinct siSUZ12 induced CCRI pathway genes in cancer and non-cancer cell lines. **A.** Heatmap of CCRI genes significantly induced by siSUZ12 (Differential Probability > 0.9) in 11 cell lines (non-cancer green, cancer blue). For cell line names corresponding to each number see (F). Blue stars in this and subsequent panels indicate cell lines in which the CCRI pathway was significantly enriched according to GSEA. **B-E** Heatmaps of subsets of the data in (A) separated by tissue type: **B.** Breast (184 non-cancer *vs*. MCF7 and MDA-MB-231 cancer), **C.** Lung (Beas-2B non-cancer *vs*. A549 cancer), **D.** Prostate (BPH-1 non-cancer *vs*. PC-3 cancer), and **E.** Another 4 cancer cell lines of pancreatic (Panc.04.03 and AsPC1), cervical (HeLa) and adrenocortical (SW-13) origin. **F.** Frequency of induced CCRI genes across all 271 CCRI genes in each cell line.

Next, we turned to the effect of PRC2 depletion on basal gene expression. Our microarray analysis in SW13 adrenal carcinoma cells did not reveal a prominent effect on immune genes until we treated with IFNγ (Fig 1), but we wondered if there might be a more prominent effect on basal expression of immune pathways in other cancer types. To decipher the key pathways affected, we used Gene Set Enrichment Analysis (GSEA) [34]. Strikingly, siSUZ12 induced the basal expression of multiple immune components in 6/8 cancer lines, of which the “cytokine/cytokine receptor interaction” (CCRI) gene set was affected in all 6 lines (Figs 4 and 5), and in 4 lines the CCRI class was the top or second ranked gene set (* in Fig P in S1 File). These findings were validated for multiple genes with two siRNAs against SUZ12 (S2 Table). Among the six cancer lines where the CCRI gene set was induced, 61% of the genes were up-regulated specifically in 1 or 2 of the cell lines (Fig Q in S1 File). Thus, PRC2 not only regulates ISGs, but its effect on the immune program in cancer cells extends to numerous cytokines and cytokine receptors, and akin to ISGs, it affects different CCRI genes in distinct cancer contexts.

In stark contrast to cancer lines, siSUZ12 did not enrich for immune gene sets in non-cancer breast, prostate and lung lines (Fig 4, Fig Q in S1 File). To expand this analysis, we used GSEA to analyze published RNAseq data from SUZ12-knockdown in human HEK-293T cells [35]. These are non-cancer-derived cells of neural origin that express viral oncogenes, but were transformed *in vitro*, independent of the immune system [36]. GSEA revealed that PRC2 depletion did not significantly affect CCRI or other immune pathways (Fig 4). Thus, PRC2 altered CCRI pathways significantly in 6/8 cancer cell lines, but 0/4 non-cancer contexts. PRC2 depletion up-regulated some CCRI genes in non-cancer derived cell lines, but fewer than in cancer cells, insufficient to achieve significance in GSEA, and the affected genes were distinct in cancer vs non-cancer lines from matching tissues (Fig 5). These data indicated a broad role for PRC2 in regulating the immune program, which is altered and expanded significantly in cancer.

### SUZ12 Knockdown Induces Genes with Bivalent Promoters

**Fig 6:**
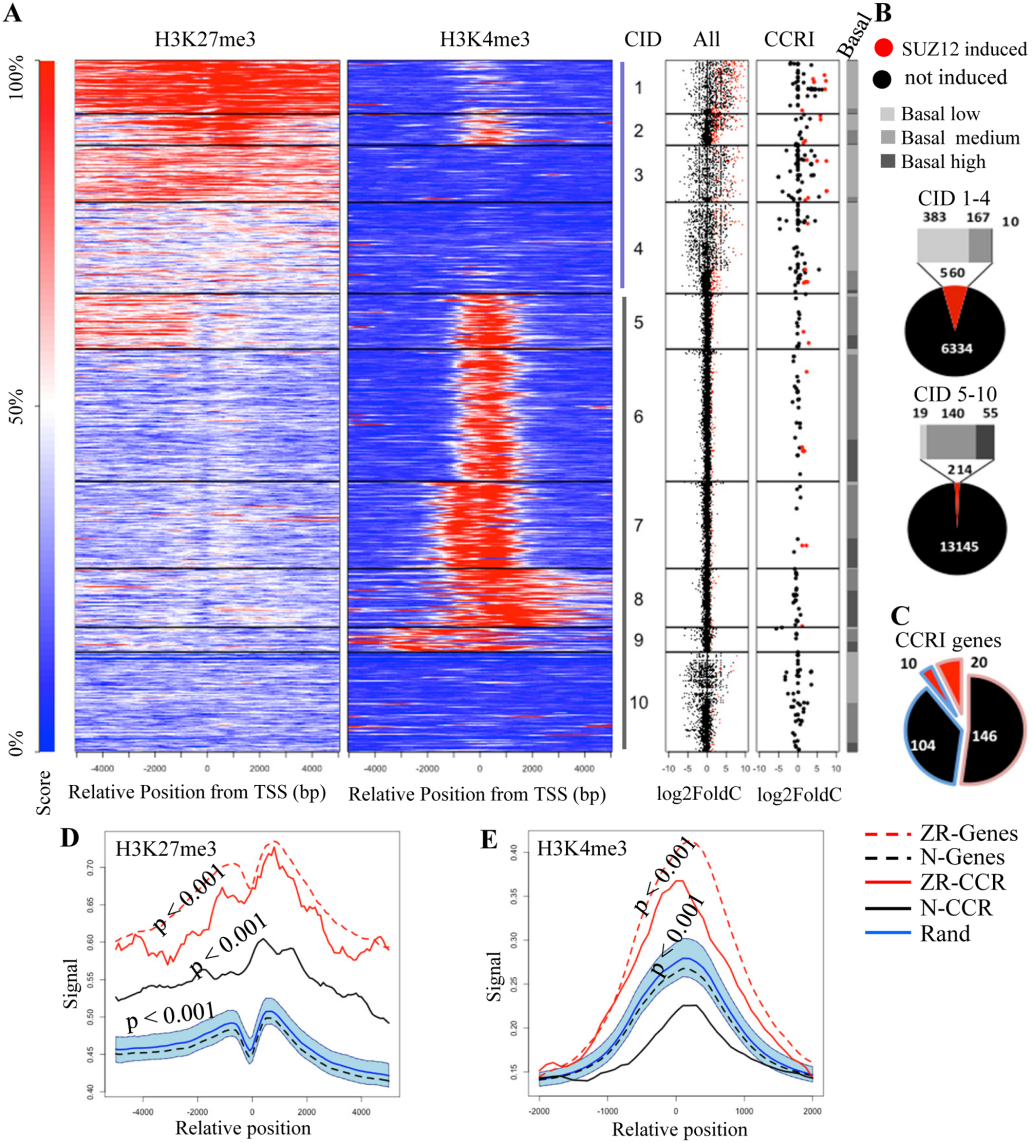
siSUZ12-induced genes are bivalent. ChIP-seq data from A549 cells was used to assess chromatin marks at siSUZ12 induced genes. **A.** Unsupervised K-means clustering was performed on H3K27me3 and H3K4me3 signals, and plotted as heatmaps around the TSS (for more details see “ChIP-seq data analysis in S1 Text, Supplementary Methods). The 10 resulting clusters (Cluster ID (**CID**) 1–10) are depicted in order of average log2Fold change in gene expression after siSUZ12 treatment as shown in the dot plots to the right. On the dot plot, all genes (left) or CCRI genes (right) show log2Fold change for each gene; red dots indicate genes significantly induced by siSUZ12 (Differential probability > 0.9). Basal expression is shown to the far right (Light grey: low; grey: medium; dark grey: high). **B.** Shows gene clusters revealed in panel A. Proportion of all genes induced by siSUZ12 (red) in CIDs 1–4, or 5–10 (as shown in the dot plots in A). **C.** Proportion of CCRI pathway genes induced by siSUZ12 (red) in CIDs 1–4 (red outline) or 5–10 (blue outline). **D.** Average H3K27me3 ChIP signal around the TSS for the indicated groups of genes (Key to the right; ZR-Genes: all SUZ12 repressed genes; N-Genes: not affected by siSUZ12; ZR-CCRI: CCRI genes that are SUZ12-repressed; N-CCRI: CCRI genes not affected by SUZ12; Rand: 1000 random gene sets, n = median of the other four gene sets). **E.** Same as (D) except for H3K4me3 ChIP signals in CIDs 1–4 (i.e. those with high H3K27me3).

To define whether PRC2-mediated repression of siSUZ12-induced genes, especially the CCRI subclass, is direct, we mined H3K27me3 ChIP-seq data available for A549 lung cancer cells, and compared it with our RNAseq data +/- siSUZ12 (Fig 6A). In parallel, we compared H3K4me3 distribution, which marks active or poised promoters. Unsupervised clustering identified 10 distinct H3K27me3/H3K4me3 patterns. Most highly induced siSUZ12 genes, including the CCRI subset were in clusters 1–4, with extensive H3K27me3 promoter coverage (Fig 6A–6D). However, most genes with high promoter H3K27me3 did not respond to siSUZ12 (Fig 6A), underscoring the importance of measuring the effect on gene expression. Notably, the average H3K4me3 signal was considerably higher across siSUZ12-responsive genes, including the CCRI subset, compared to genes that were unaffected by siSUZ12 or 1000 random genes sets (Fig 6E). Thus, genes that are induced following PRC2 disruption have a bivalent H3K27me3/H3K4me3 signature, and siSUZ12-induced CCRI genes are typical of this group.

### Small Molecule EZH2 Inhibitors Augment Multiple Immune Pathways

Our data raise the possibility that drugs that inhibit PRC2 might boost immune pathway activation in cancer cells. When four cancer cell lines were treated with 2 μM of GSK343 [37] or UNC1999 [38], either drug markedly reduced H3K27me3 levels (Fig R in S1 File). RT-PCR analysis revealed that of 8 SUZ12-repressed ISGs (ZR-ISGs) in 4 cell lines (32 cases) UNC1999 augmented IFNγ-responsiveness in 30 cases (94%) and GSK343 did so in 15 (50%) (Fig S in S1 File). Treatment had no effect on non-ISGs such as *PITX2* or *HPRT* or on the mRNA or protein levels of IRF1, a PRC2-independent ISG (Figs R, S in S1 File). UNC1999 induced mRNAs to a greater extent than GSK343 despite apparently similar effects on bulk H3K27me3 levels. This may reflect subtle differences in the kinetics and/or total amount of H3K27me3 depletion at specific enhancers or promoters, which would not revealed by Western analysis of total H3K27me3. Irrespective, these data show that EZH2 inhibitors, like siSUZ12, augment mRNA induction of PRC2-repressed ISGs.

**Fig 7:**
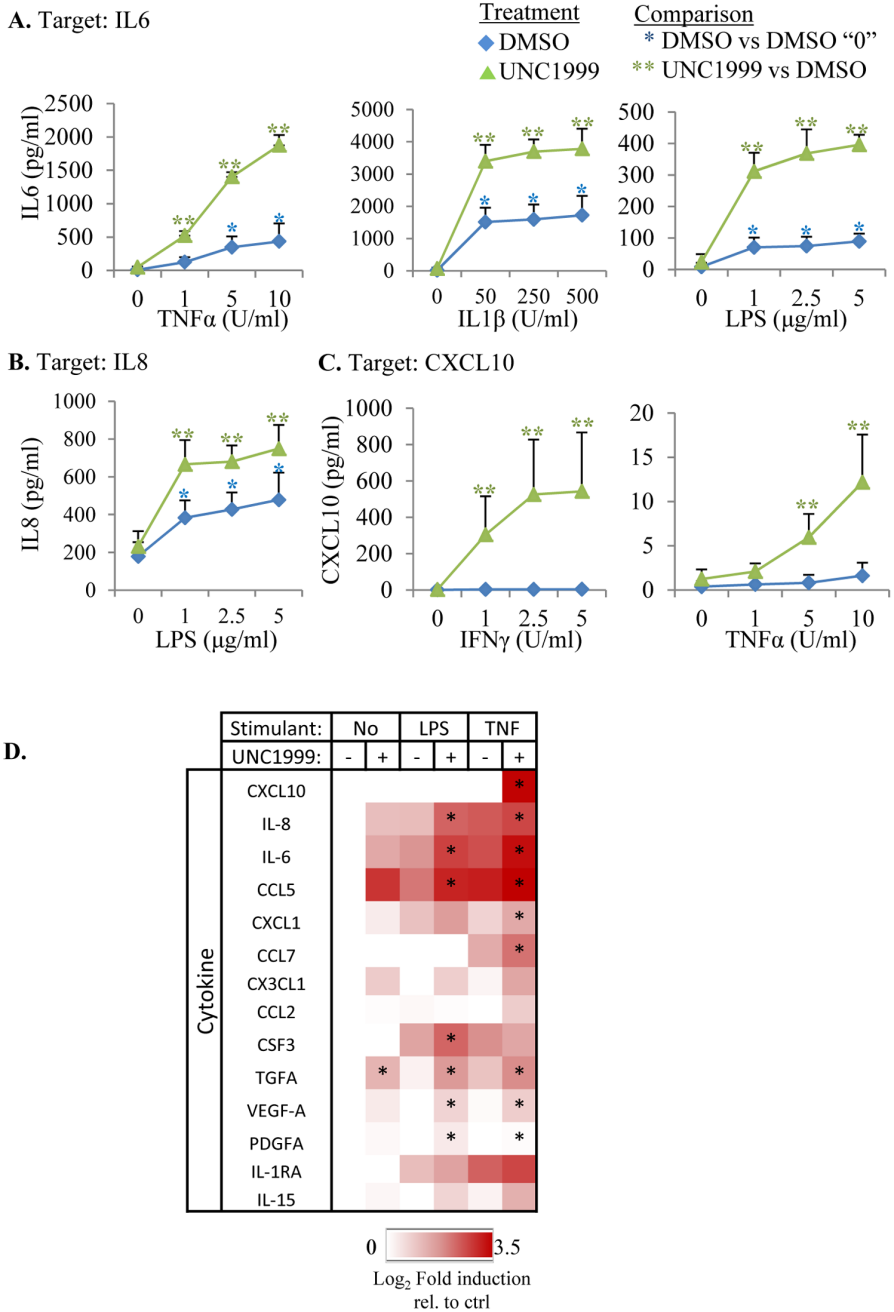
Pharmacological inhibition of PRC2 boosts multiple immune pathways. A549 cells treated with UNC1999 (green) or DMSO (blue) were stimulated with the indicated concentrations of TNFα, IFNγ, IL1β or LPS for 24 h and ELISA was performed for secreted IL6 **(A)**, IL8 **(B)** and CXCL10 **(C)**. Asterisks indicate significant effects (P< 0.05; n = 3; ANOVA followed by Fisher test) according to the indicated comparisons. (**D**) A549 cells treated with vehicle (-) or UNC1999 (+) as indicated were exposed to No immune stimulus, 5μg/ml LPS, or 10U/ml TNFα for 24 h, and the concentration of secreted cytokines assessed using a Multiplexing Laser Bead assay. Heat map represents the log_2_ average fold induction from three biological replicates compared to vehicle with No immune stimulus. Asterisks indicate significant effects (P< 0.05; n = 3; ANOVA followed by Fisher test) between-/+ UNC1999 for each stimulus.

Finally, we assessed whether PRC2 inhibition boosts cytokine induction in response to other immune signals and whether these effects are observed at the protein level. For this, we treated A549 lung cancer cells with TNFα, IL1β, *E*.*coli* lipopolysaccharide (LPS), as well as IFNγ, and assessed secretion of the cytokines CXCL10, IL6 and IL8. Unlike mRNA induction, PRC2 inhibition alone did not enhance protein levels but when cells were exposed to one of the four immune signals, siSUZ12 or UNC1999 augmented cytokine protein induction (Fig 7A–7C, Fig T in S1 File, S2 Table). These data are in line with the well-established, post-transcriptional regulation of cytokine production to prevent adverse immune reactions, such as chronic inflammation. For example, in addition to gene transcription (within the direct control of PRC2), cytokines are also tightly regulated at the level of mRNA stability and/or translation through motifs such as the AU-Rich Element (ARE), Constitutive Decay Element (CDE), Coding Region Determinant of Instability (CRD) and several others [39]. Our results suggest that PRC2 regulates the transcriptional component, but not the post-transcriptional control, which requires an additional signal from the immune system. To expand this analysis we used a multiplex laser bead assay to assess the effect of UNC1999 on LPS or TNFα induced production of multiple cytokines in A549 lung cancer cells. Fourteen of the tested proteins were induced in at least one condition, and for 10/14 PRC2 inhibition significantly boosted basal, LPS-induced, and/or TNFα-induced cytokine production, while in 4 cases there was a trend towards augmented cytokine induction (Fig 7D). Together, our combined gene and protein analysis demonstrate that PRC2 inhibition is a potent strategy to augment both cytokine responsiveness (Figs 3 and 7) and cytokine secretion (Fig 7, Fig T in S1 File) in cancer cells.

## Discussion

PRC2 is thought to influence cancer progression through effects on division, senescence, survival and stemness [5–7]. Our data now reveal a profound and cancer-selective role for PRC2 in repressing multiple immune pathways. These data have important implications both for our understanding of the role of PRC2 in tumorigenesis, and for the mechanism of action and potential efficacy of EZH2 inhibitors in treating human cancer.

IFNγ is critical for immune surveillance [13–20], and depleting PRC2 reactivated numerous ISGs. Moreover, PRC2 also repressed multiple cytokines and cytokine receptors. Thus, cancers co-opt PRC2 to alter both the cytokine cocktail to which they and cells in their environment are exposed, and to alter cytokine gene targets. An EZH2 inhibitor rescued ISG expression and augmented cytokine secretion in response to several immune signals. While IFNγ blocks tumorigenesis in multiple scenarios, many other cytokines have context dependent positive and/or negative effects on cancer (S2 Table)[3,22]. Indeed, cytokines regulate many processes that affect tumorigenesis, such as immunity, proliferation, survival, senescence, invasiveness, angiogenesis, and drug resistance [22]. There is excitement over the potential of EZH2 inhibitors to treat cancer, and their ability to augment protective cytokine pathways (e.g. IFNγ) could boost efficacy. Equally, however, the release of some cytokines could be detrimental. Thus the result of the putative “immune bomb” detonated upon PRC2 inhibition is difficult to predict. Moreover, in view of the distinct immune genes targeted by PRC2 in different cancer types, the effects are likely to be highly cancer-specific. Our results should stimulate studies in syngeneic models to deduce the context-specific effects of PRC2 inhibition on different cancers, in the presence or absence of an immune system. It will also be critical to track immune parameters in clinical trials on EZH2 inhibitors, as the effect of these drugs on cytokine production and immune cell infiltration could have dramatic effects on outcome.

A few reports have linked PRC2 to individual immune genes [40–45], and it regulates the differentiation of normal immune cells [40,45,46], but the alteration and augmentation of this function to a considerable extent in cancer cells is unexpected. Previous work assessed the basal effects of PRC2 inhibition, which would miss the prominent effect on gene induction by immune signals such as IFNγ, TNFα, IL1β, or LPS reported here. Moreover, many prior studies on PRC2 targets focused on non-cancer cells (especially embryonic stem cells), and as our results show, the prominent effect on immune genes is cancer-selective. Studies on PRC2 in cancer cells have generally focused on recruitment rather than effects on transcription, and as we note here, only a subset of PRC2/H3K27me3 marked targets are alleviated upon PRC2 inhibition. Immune and other gene targets that had the bivalent H3K27me3/H3K4me3 mark were induced upon PRC2 depletion, which is consistent with other analyses [47]. A recent study linked a non-enzymatic effect of EZH2 to regulation of the innate response to virus infection [48]. However, we observe a direct link with H3K27me3 and a potent effect of pharmaceutical inhibitors, indicating that methyl transferase activity is critical for PRC2 regulation of cytokine pathways in cancer cells.

Our results here and in a complementary study [12] suggest that PRC2 directly represses many ISGs and CCRI genes. While we did not examine PRC2 subunit recruitment in this paper, we provide extensive data showing that the genes rescued by PRC2 depletion are marked with H3K27me3. This work included ChIP-qPCR analysis of 25 ISGs (Fig E in S1 File), genome wide ChIP-chip (Fig 2, Figs F and G in S1 File), and analysis of ENCODE Chip-seq data (Fig 6). In each of these analyses, genes affected by PRC2 exhibited H3K27me3, whereas most genes that were unaffected lacked this mark. Since PRC2 is the only enzyme that deposits this mark, it is logical to conclude that these loci are direct PRC2 targets. Moreover, in parallel work we find that SUZ12 and EZH2 bind multiple locations across the *CIITA* locus in a pattern almost indistinguishable from that of H3K27me3 [12]. Altogether, these extensive data provide a strong case for the notion that PRC2 directly targets ISG and cytokine pathway genes. Not every gene that is marked with H3K27me3 is induced upon PRC2 depletion. Repression of these genes in the absence of PRC2 may be mediated by redundant repressive complexes such as PRC1 [49,50] and/or the absence of active chromatin marks. Indeed, our analysis of Chip-seq data revealed that CCRI genes that respond to PRC2 depletion are marked not only with H3K27me3, but also with H3K4me3 (Fig 6). Thus, “bivalent” promoters are responsive to PRC2 inhibition, which is consistent with literature indicating that active chromatin marks are required for gene activation when repressive chromatin marks are removed [47,51,52].

Our work reveals an intriguing cancer-selective role for PRC2 at cytokine pathway genes, thus raising the issue as to how this functional shift is achieved. While the level of PRC2 subunits was elevated in some cancer cell lines this was variable (Fig H in S1 File), and we thus favor the hypothesis that it is the mechanism of PRC2 recruitment that is altered in cancer cells to down-regulate ISGs and CCRI genes. Many factors can influence PRC2 recruitment [53] such as Jarid2 [40,54,55], ATRX [56]), PHF1/PCL1 [57], KDM2B plus PCGF1/PRC1 [58], and long non-coding RNAs [35,59,60]. Elucidating whether these and/or other mechanisms influence PRC2 recruitment to cytokine pathway loci is beyond the scope of the current work, but will be an important topic for future research. SUZ12-depletion induced some CCRI genes in non-cancer lines (Fig 5). Recent work in a non-human cell line also suggested a potential role for PRC2 in immune modulation in the gut [61]. It will be important to discover how this normal role is expanded and altered in cancer as it may reveal strategies to modulate this activity in a tumor-specific manner.

Blocking PRC2 rescued IFNγ induction even in the absence of BRG1, normally essential for ISG activation [8,9]. Indeed, whereas only 2% of all genes were affected by BRG1 or PRC2, almost 90% of ISGs were BRG1-dependent and/or PRC2-repressed, and H3K27me3 was prominent at ISGs genome-wide. This result exposes a new immune role for the antagonistic Trithorax (e.g. SWI/SNF) and Polycomb (e.g. PRC2) regulators. SWI/SNF defects and/or PRC2-induction are common in human cancer [5,6], thus our results raise the hypothesis that multiple cancer types could modify immune signaling and possibly immune surveillance through such perturbations.

While the current work exposes a genome wide role for SWI/SNF and PRC2 in affecting ISG responsiveness, it does not reveal the underlying mechanism of their antagonism. However, in parallel work, we have obtained insight into this issue by focusing on the *CIITA* locus [12]. Like many other ISGs, either BRG1 reconstitution or PRC2 depletion in BRG1-deficient cells facilitates IFNγ responsiveness at this locus and both factors are recruited to the locus. BRG1 is targeted to a remote upstream enhancer, while PRC2 and H3K27me3 exhibit an undulating pattern across the locus, peaking at promoter and inter-enhancer locations. Notably, reconstituting cells with BRG1 did not reduce SUZ12 or EZH2 binding across the locus, suggesting that BRG1 does not compete with PRC2 for chromatin binding, a mechanism proposed before to explain SWI/SNF-PcG antagonism [62]. The most obvious effect of reintroducing BRG1 was the addition of H3K4me1 and H3K27ac marks at the remote -50 kb enhancer where BRG1 is recruited, which are well known to poise enhancers. Strikingly, depleting PRC2 caused induction of the same marks, arguing that SWI/SNF and PRC2 do not directly antagonize each other, but rather antagonize the recruitment and/or action of histone acetyl and methyl transferases required to poise the locus for IFNγ responsiveness. It seems likely that this mechanism may operate at other ISGs, and additional genome wide analyses will be required to test this notion.

PRC2-depletion augmented specific cocktails of ISG and CCRI genes in distinct cancers. This observation suggests how a single factor could sculpt differential immune responses in each tumor. To escape elimination by the immune system, tumors first enter a period of equilibrium followed by escape [63]. PRC2 could regulate both stages, thus EZH2 inhibitors should be assessed in cancer prevention as well as therapy of advanced tumors. Some tumors have inactivating PRC2 mutations [6,64,65] and must utilize a PRC2-independent mechanism to modify the immune response. Conceivably, these inactivating mutations could result in up-regulation of cytokines that promote tumorigenesis. We studied cell lines from solid tumors, all with intact functional PRC2, and inhibiting the repressor boosted ISG-induction in 7/8 lines, and CCRI gene induction in 6/8 lines. Of the two cancer lines where CCRI gene-set induction did not reach significance, one of the lines (SW-13) lacks SWI/SNF activity, providing an alternative means to quench immune pathways and possible anticancer immunity. Notably, PRC2 repressed multiple ISGs in both these lines. In summary, SWI/SNF and PRC2 regulate a broad range of cytokine pathway genes in human cancer cells.

## Supporting Information

S1 File Supplementary Figures A-T. (PDF)

S1 Table Type and frequency of gene classes identified through RNAseq analysis. (XLSX)

S2 Table Summary of how various PRC2-repressed cytokine and cytokine receptor gene targets were validated at the RNA and/or protein level. (XLSX)

S3 Table Antibodies. (XLSX)

S4 Table Primers. (XLSX)

S1 Text Supplementary Text Includes Supplementary Results (more details on the microarray and RT-PCR data in Fig 1 and Supp Figs A-D, as well as a detailed description of RNAseq data validation), and Supplementary Methods.
(DOCX)
