# Supplementary material for: Cancer Cells Hijack PRC2 to Modify Multiple Cytokine Pathways
Source: PLoS One. 2015 Jun 1;10(6):e0126466. doi: 10.1371/journal.pone.0126466 (PMC4450877; doi:10.1371/journal.pone.0126466)
Supplement: S1 File — (PDF) [file pone.0126466.s001.pdf]

(A)

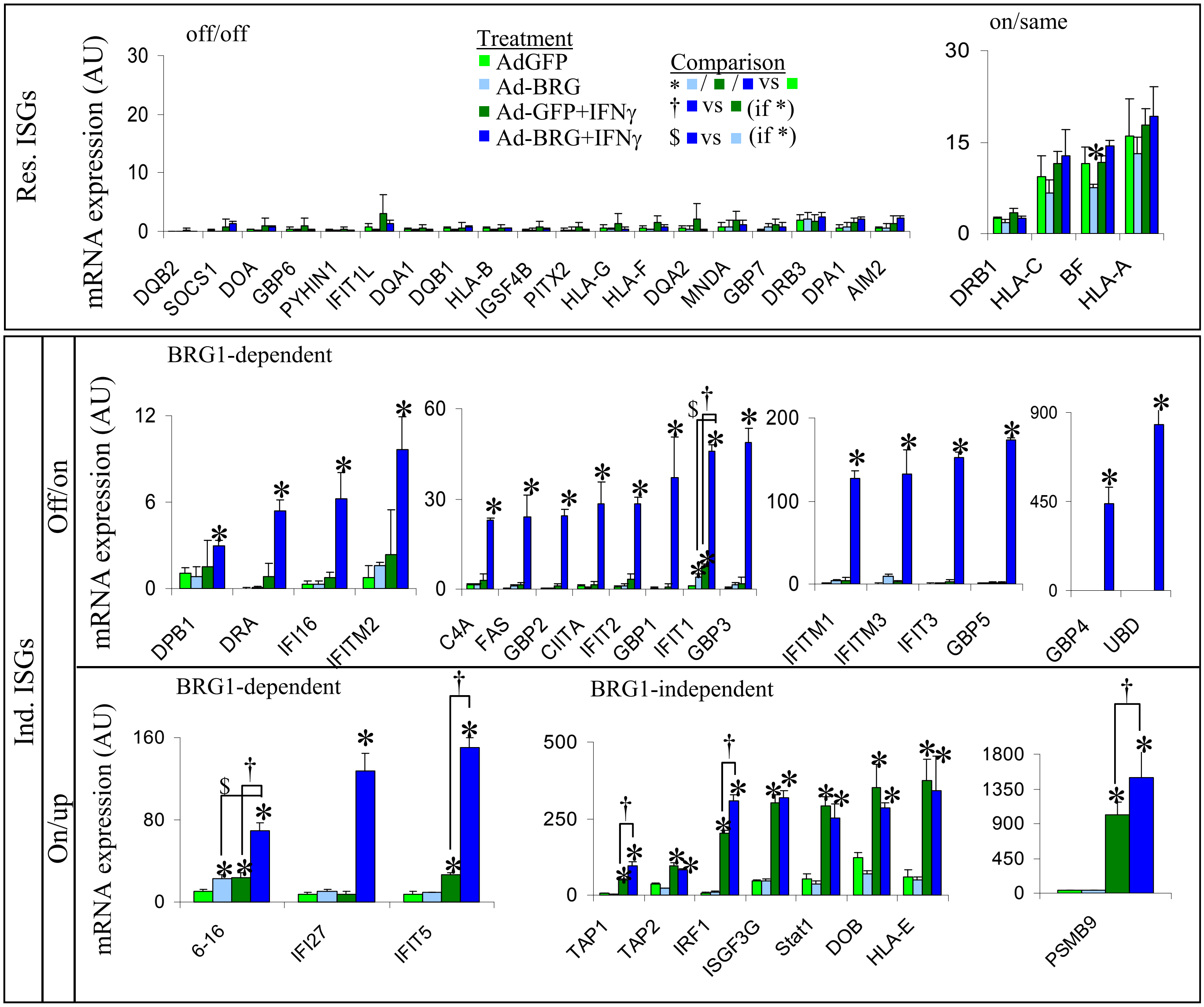

(B)

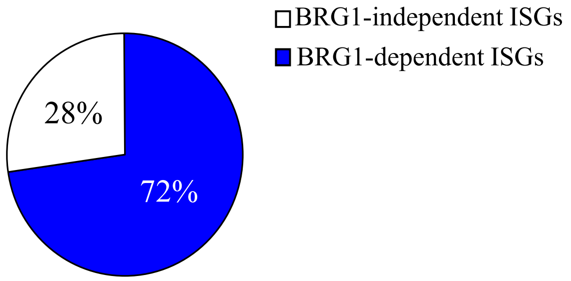

Supplementary Figure A. Multiple ISGs are BRG1-dependent. **(A)** Real-time PCR analysis of 55 ISGs in SW-13 cells transduced with Ad-GFP or Ad-BRG1 for 24 hours then left untreated or treated with IFNγ for 6 hrs. Gene expression was normalized to Tubulin and presented in arbitrary units (AU). Genes with AU of < 2 are considered silent (off). Values are mean ($n \geq 3$) +/- S.D. *, †, and $ indicate significantly changed genes ($P < 0.05$, ANOVA followed by Fisher test) according to the indicated comparisons (key). ISGs are presented according to responsiveness to IFNγ into resistant (Off/off, On/same) or induced genes (Off/on, On/up), and induced genes were further sorted according to BRG1 dependency. **(B)** Pie graph summarizing BRG1 dependency of responsive ISGs (Off/on and On/up).

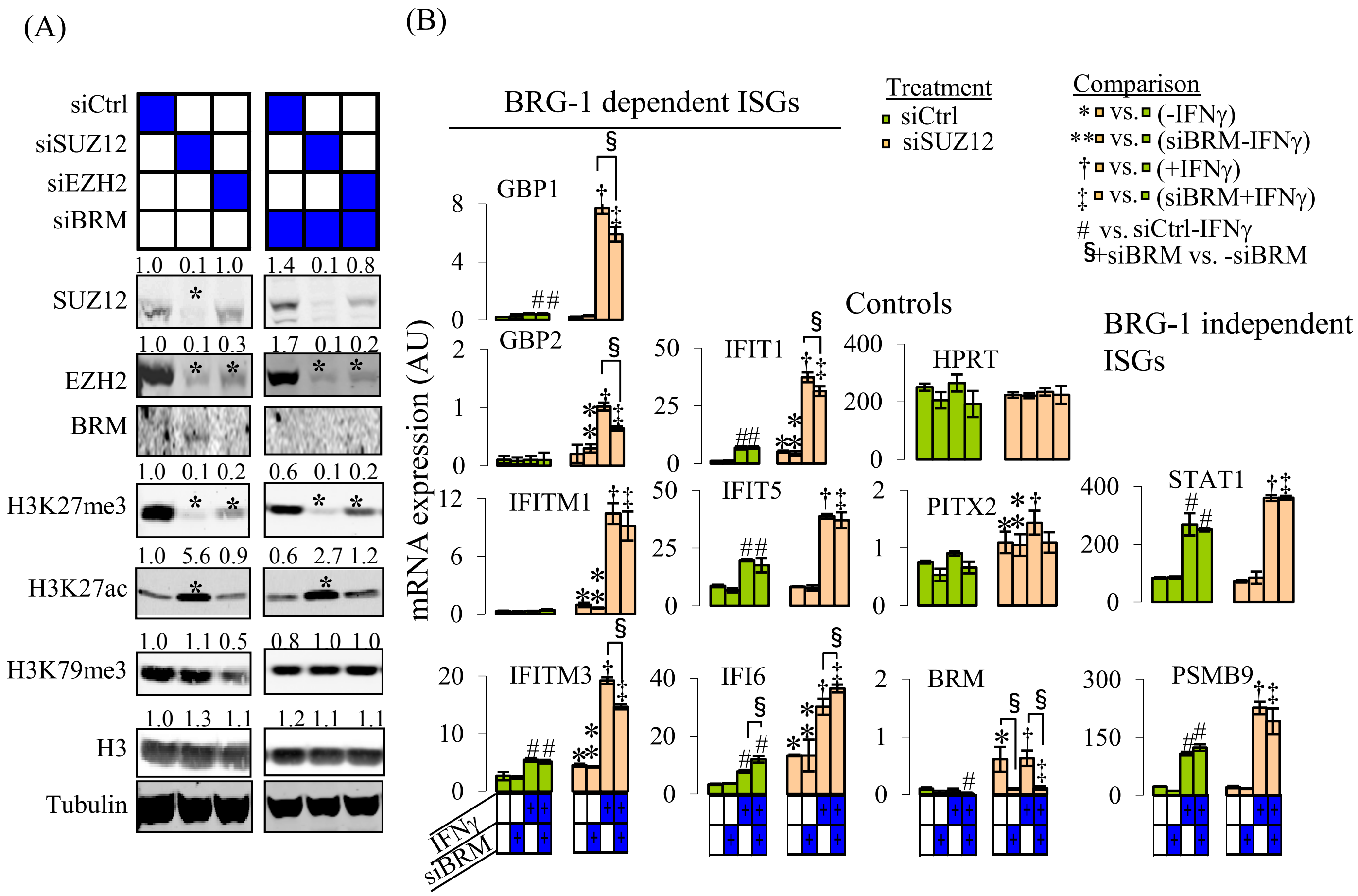

Supplementary Figure B. PRC2-depletion rescues responsiveness of BRG1-dependent ISGs in BRG1-deficient cells. **(A)** Representative Western blots of the indicated factors and histone modifications. Analyses were performed using lysates of SW-13 cells treated with siCtrl or siSUZ12 alone or in combination with siBRM. siRNA treatments are indicated by blue squares. Data were reproduced at least twice and protein levels quantified relative to Tubulin. Asterisks indicate significant changes compared to siCtrl group ($p<0.05$, ANOVA followed by Fisher test). **(B)** Real-time PCR analysis of the indicated genes in SW-13 cells transfected with siCtrl or siSUZ12 alone or in combination with siBRM and left untreated or exposed to IFNγ for 6 hrs. Treatments are indicated in blue in the beneath table. Gene expression was normalized to Tubulin and presented in arbitrary units (AU). Values are mean ($n \geq 3$) +/- S.D. *, **, †, ‡ indicate significant changes ($p<0.05$, ANOVA followed by Fisher test) according to the indicated comparison (key).

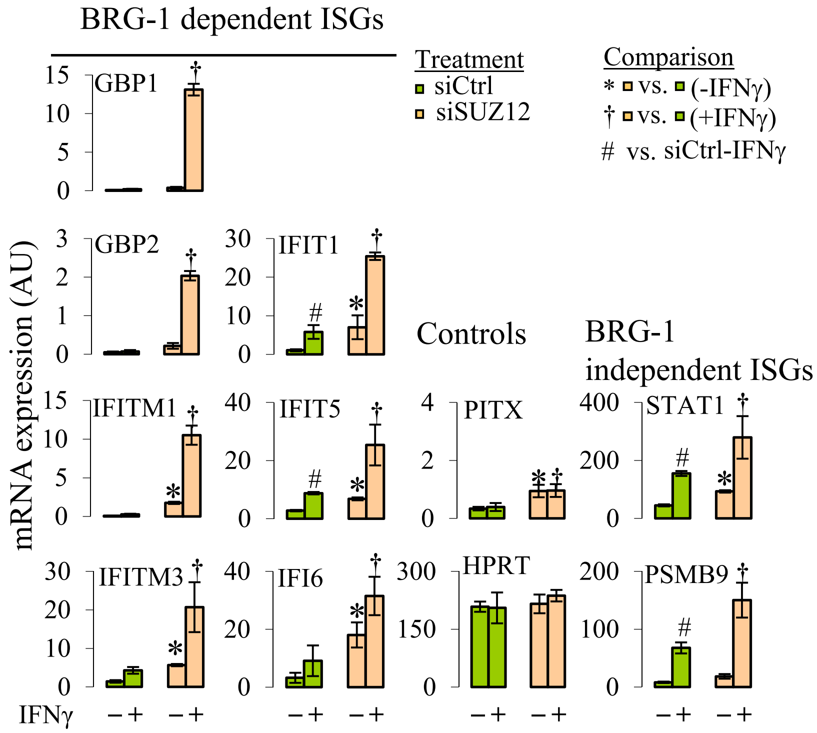

Supplementary Figure C. Rescue of ISGs responsiveness by siSUZ12 is not due to an off target effect. Real-time PCR analysis of the indicated genes in SW-13 cells transfected with siCtrl or siSUZ12 and left untreated or exposed to IFNγ for 6 hrs. Gene expression was normalized to Tubulin and presented in arbitrary units (AU). Values are mean ($n \geq 3$) +/- S.D. #, * and † indicate significant changes ($P < 0.05$, ANOVA followed by Fisher test) according to the indicated comparisons (key).

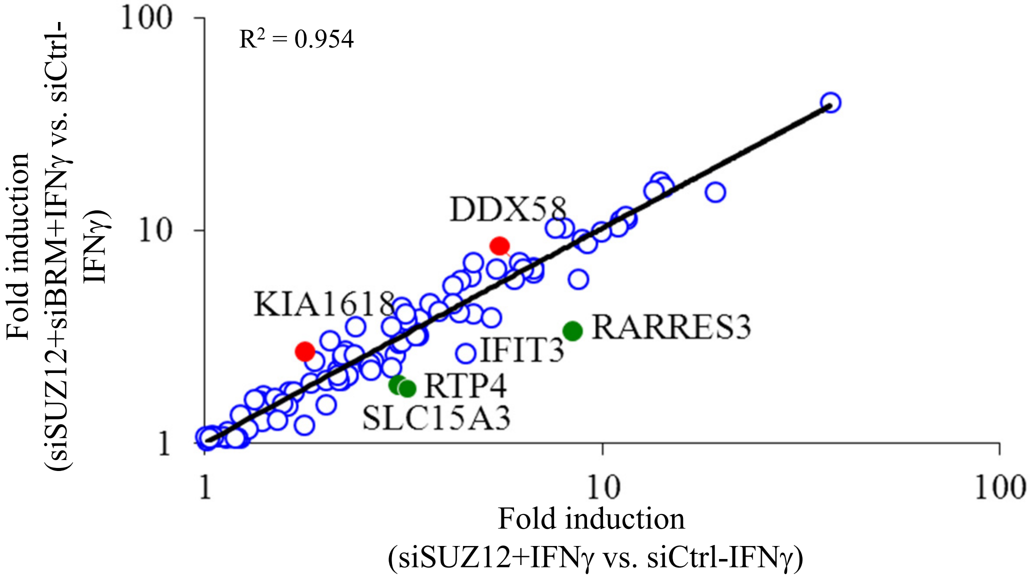

Supplementary Figure D. BRM depletion has minimal effect on the expression levels of SUZ12-repressed ISGs. Microarrays were performed with RNA from cells exposed to siSUZ12 or siSUZ12 + siBRM and left untreated or exposed to IFNγ. For the vast majority of genes fold induction by IFNγ after SUZ12 knockdown was similar (<1.5 fold difference) whether BRM was depleted or not (blue circles), and very few ISGs were up- (red) or down-regulated (green) ≥1.5 fold.

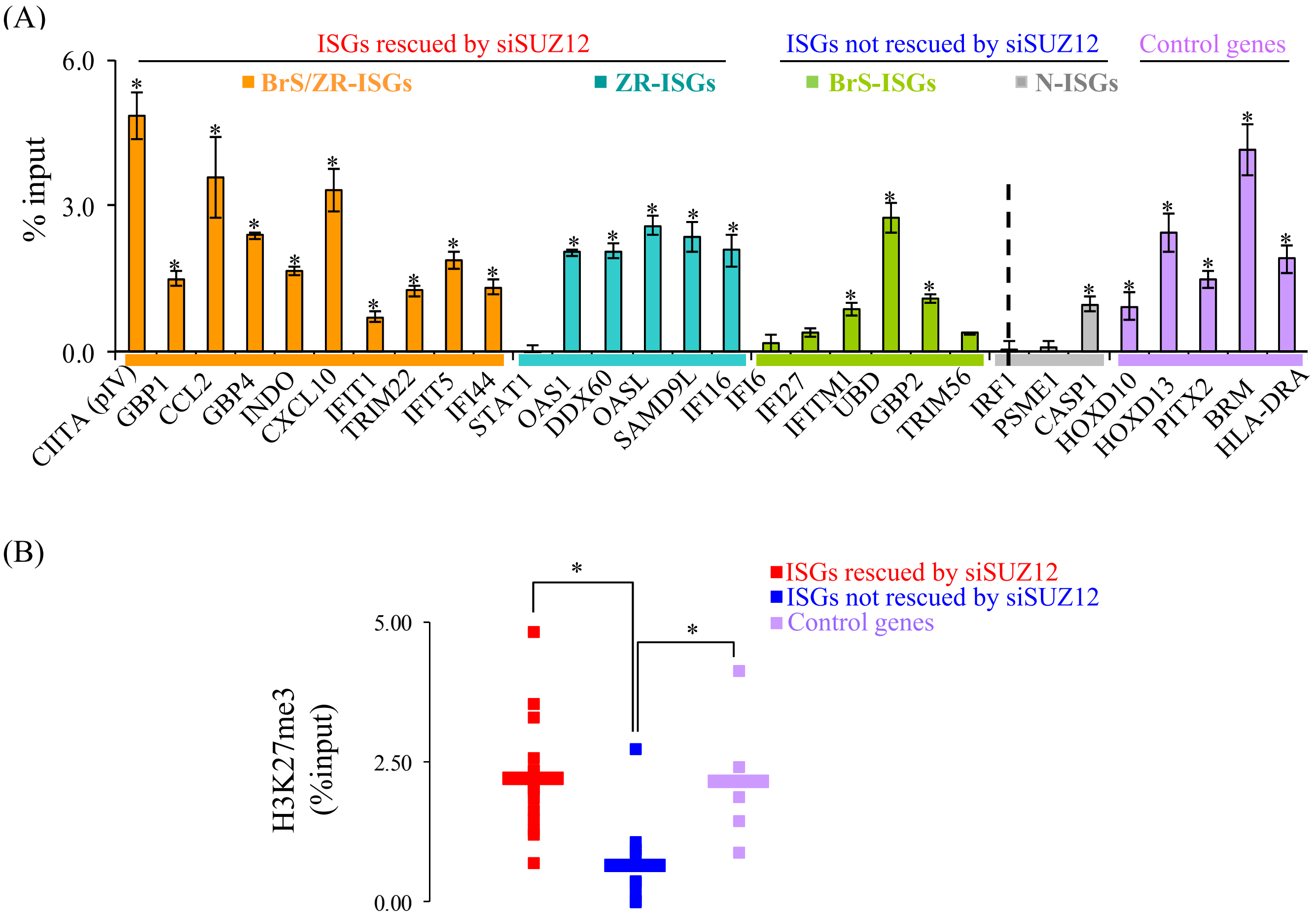

Supplementary Figure E. siSUZ12-rescued ISGs are direct PRC2 targets. (**A**) ChIP-qPCR on chromatin from SW-13 cells was performed to assess basal H3K27me3 levels at selected SUZ12-dependent and independent ISG promoters. Data are % of input DNA. Values are mean ($n \geq 3$) +/- SD. * indicates ISG promoters with significant basal H3K27me3 compared to IRF1 promoter (dashed line; $p<0.05$, ANOVA followed by Fisher test). (**B**) Average H3K27me3 levels at SUZ12-rescued ISGs or positive control gene promoters (shown in A) are significantly higher (* $p<0.0005$, Student's t test) than H3K27me3 levels at SUZ12-independent ISGs. *Gene classes:*

*1.BrS/ZR-ISG, **BR**G1-**S**timulated and SU**Z**12-**R**epressed **I**FNγ **S**timulated **G**ene (**ISG)***

*2.ZR-ISG, SU**Z**12-**R**epressed **ISG***

*3.BrS-ISGs, **BR**G1-**s**timulated **ISG***

*4.N-ISG, **N**ot affected by SUZ12, but an **ISG***

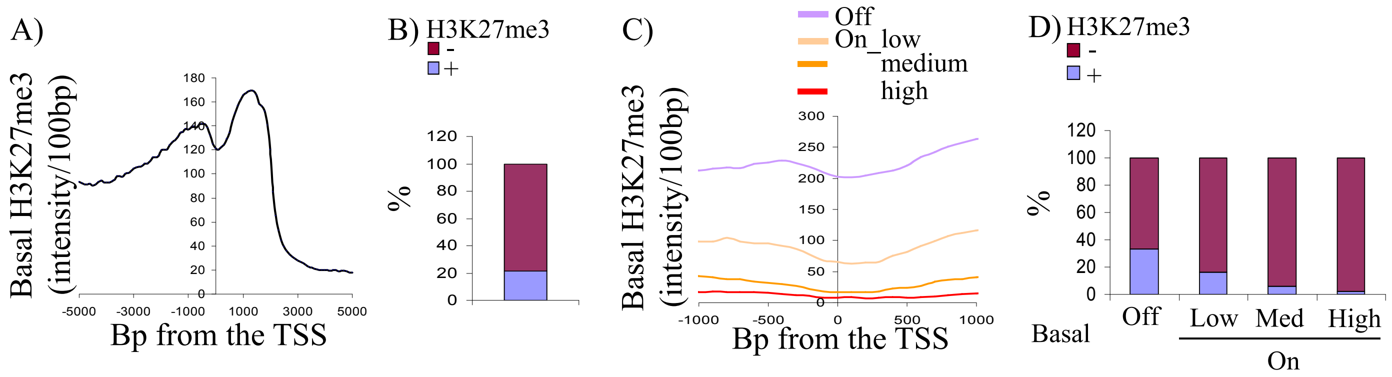

Supplementary Figure F. Promoter H3K27me3 anti-correlates with gene expression. ChIP-chip was performed using chromatin from SW-13 cells and a genome-wide promoter array. **(A)** ChIP-chip signal intensity in 100 bp bins +/-5 kb of the TSS of All genes. **(B)** Histogram of the percentage of All gene promoters positive or negative for H3K27me3. **(C)** ChIP-chip signal intensity as in (A) but grouped according to basal expression. **(D)** Histogram of the percentage of H3K27me3 positive and negative promoters among basally silent (Off) or active genes expressed at the indicated level.

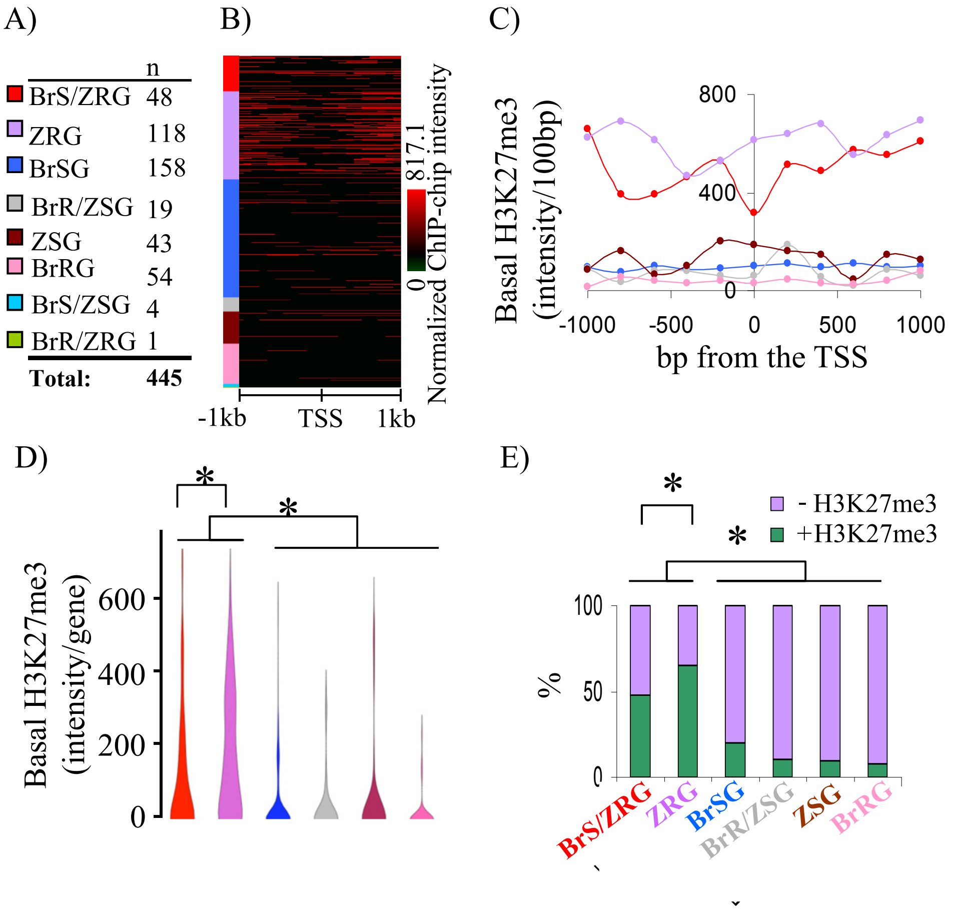

Supplementary Figure G. H3K27me3 is enriched at siSUZ12-induced genes. (A) Color-code for gene classes in (B)-(D). **(B)** Heatmap shows basal H3K27me3 ChIP-chip signal within +/-1 kb of the TSS of 445 differentially expressed genes with annotated TSSs in siSUZ12 or AdBRG1 treated cells presented in Fig 1A. **(C)** ChIP-chip signal intensity per 100 bp bins within +/-1kb of the TSS of each gene class. **(D)** Violin plot shows the average normalized ChIP-chip signal across 100bp bins +/-1 kb of the TSS of each gene class (* P < 0.05; Mann Whitney test). **(E)** Histogram shows the percentage of H3K27me3 positive and negative promoters in each indicated gene class. *: significantly higher % of H3K27me3 positive genes between the indicated groups (P < 0.05, Fisher exact test).

Gene classes:

*1.BrS/ZRG, **BR**G1-**S**timulated and SU**Z**12-**R**epressed **G**ene*

*2.ZRG, SU**Z**12-**R**epressed **G**ene*

*3.BrSG, **BR**G1-**s**timulated **G**ene*

*4.BrR/ZSG, **BR**G1-**R**epressed and SU**Z**12-**S**timulated **G**ene*

*5.ZSG, SU**Z**12-**S**timulated **G**ene*

*6.BrRG, **BR**G1-**R**epressed **G**ene*

*7.BrS/ZSG, **BR**G1-**S**timulated and SU**Z**12-**S**timulated **G**ene*

*8.BrR/ZRG, **BR**G1-**R**epressed and SU**Z**12-**R**epressed **G**ene*

B)

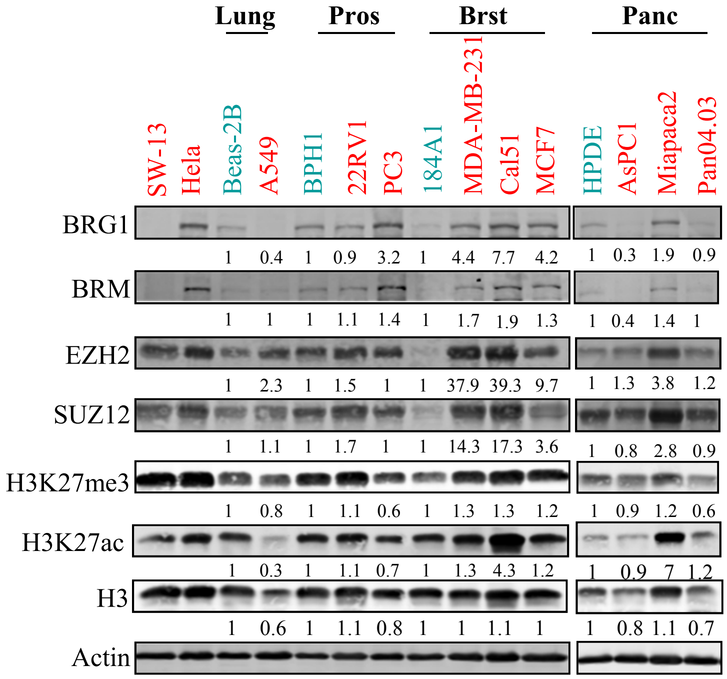

B)

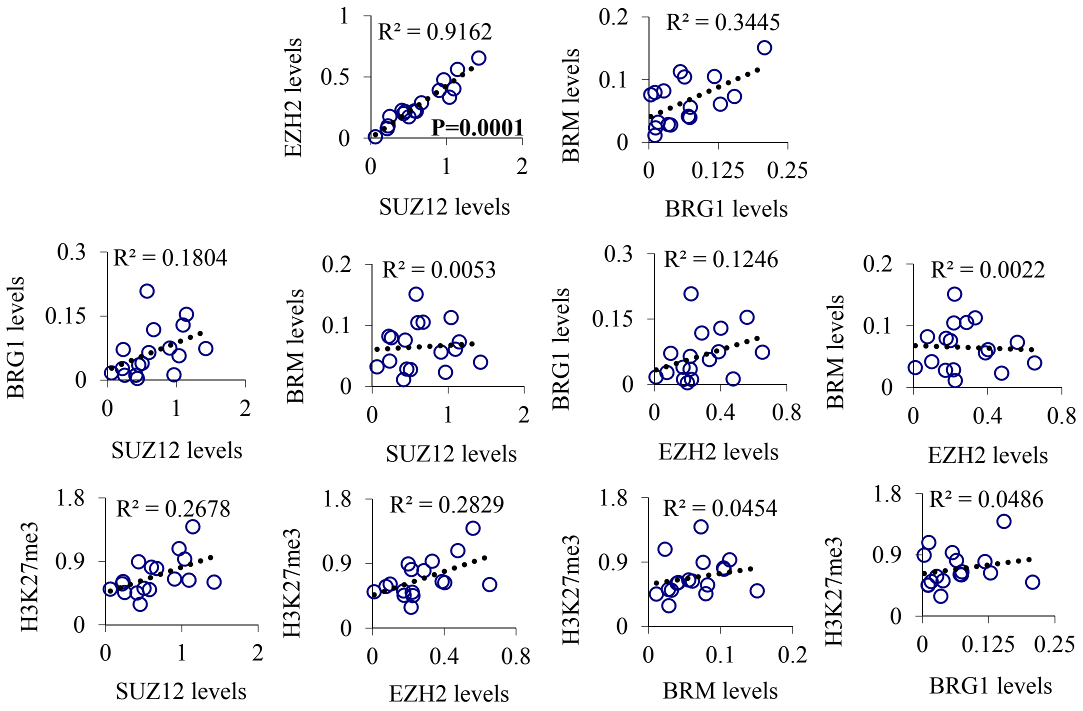

Supplementary Figure H. Basal expression of SWI/SNF and PRC2 subunits and H3K27me3 in a panel of non-cancer and cancer derived cell lines. (A) Western blots show the basal expression of the indicated proteins. Cells were segregated based on their tissue of origin, with non-cancer-derived control cells first from the left (green) followed by cancer cells (red). Protein levels were normalized to Actin. Normalized protein expression levels in cancer cells are presented below blots as fold above the levels in the non-cancer derived cells of the same tissue origin. **(B)** Regression analysis indicated a correlation between the PRC2 subunits EZH2 and SUZ12, but not other protein pairs.

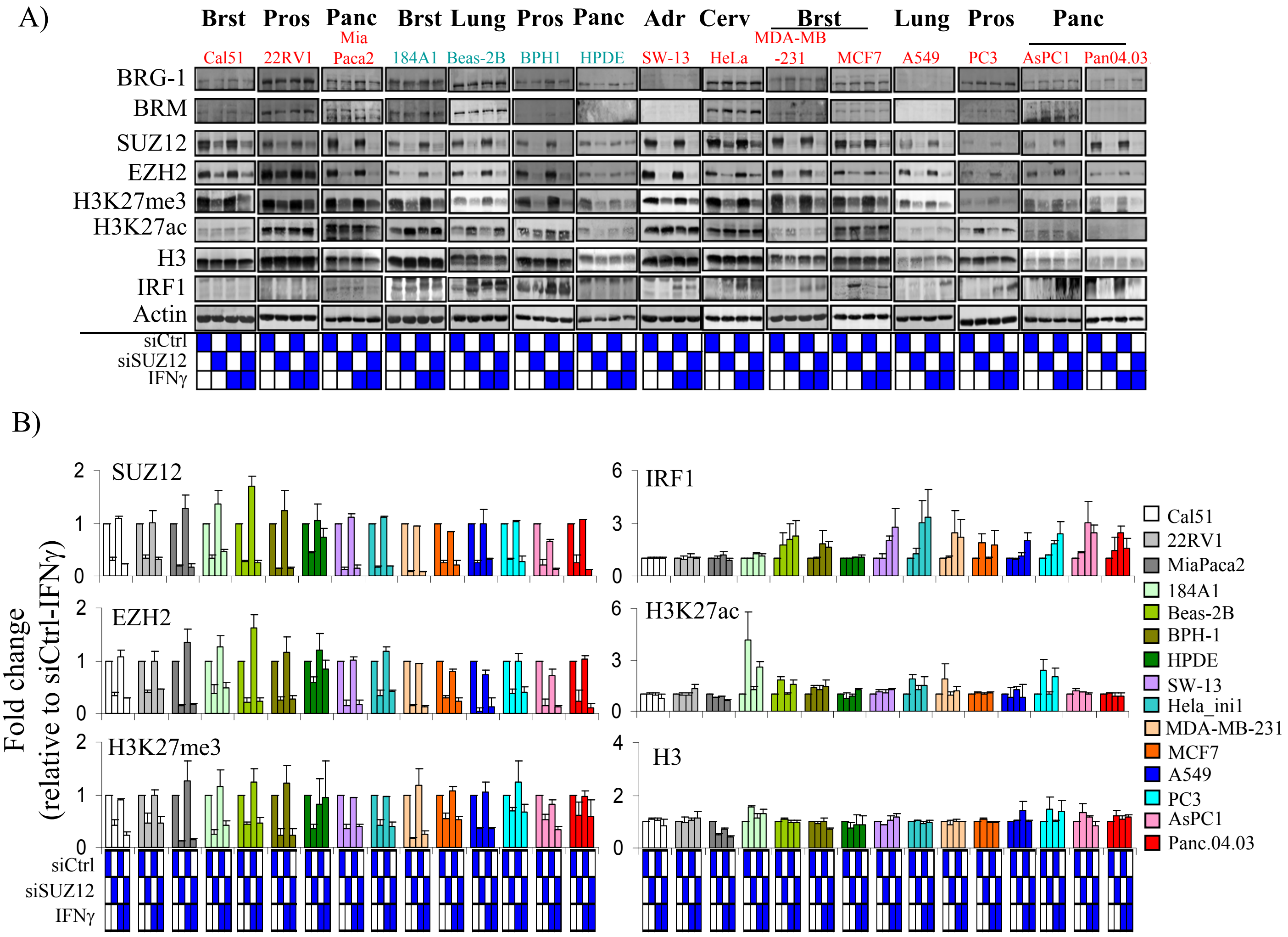

Supplementary Figure I. siSUZ12 inhibits H3K27 tri-methylation in human non-cancer and cancer derived cell lines. (A) Western blot analyses of the indicated SWI/SNF and PRC2 subunits, IRF1 and total and modified histone H3 were performed using lysates of non-cancer (green) and cancer (red) derived cell lines. Cells were transfected with siCtrl or siSUZ12 and left untreated or stimulated with IFNγ for 6 hrs. Treatments are indicated by blue squares below the blots. **(B)** Protein expression was quantified relative to Actin and changes in protein levels were expressed as fold above the siCtrl minus IFNγ control. Values are mean (n=2) +/- range.

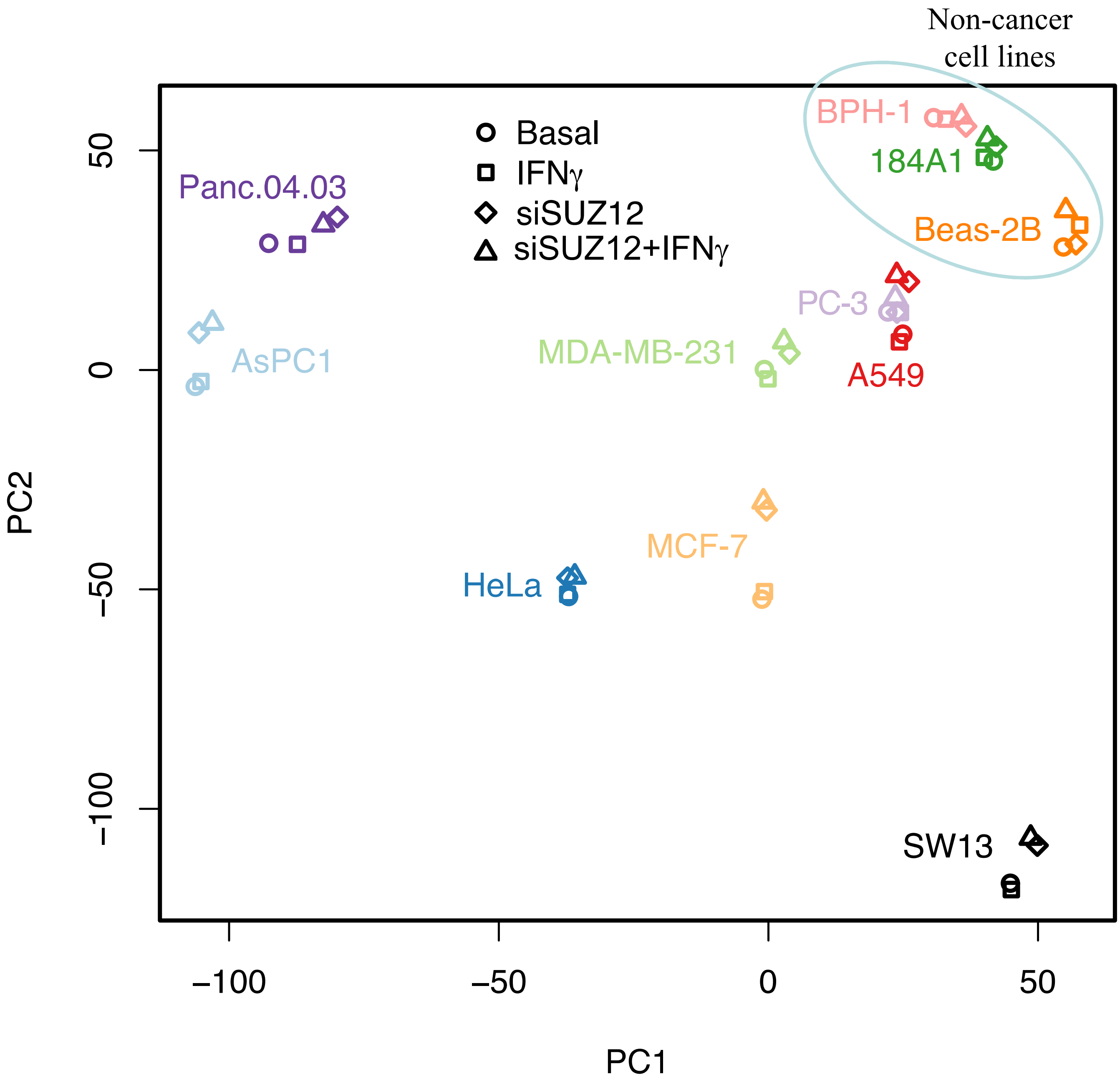

Supplementary Figure J. Principal Component Analysis (PCA) of RNAseq data. 44 samples of 11 cell lines and 4 treatments are shown in the 2D plane spanned by their first two principal components based on the top 500 genes with the largest variance**.** The overall effects of covariates are from differences between cancer and non-cancer and from differences between cell lines. No batch effects were detected. The original tissues of the 11 cell lines are: breast (184, non-cancer; MCF7 & MDA-MB-231, cancer), lung (Beas-2B, non-cancer; A549, cancer), prostate (BPH-1, non-cancer; PC-3, cancer), pancreas (Panc.04.03 & AsPC1, cancer), cervix (HeLa, cancer) and adrenal cortex (SW-13, cancer).

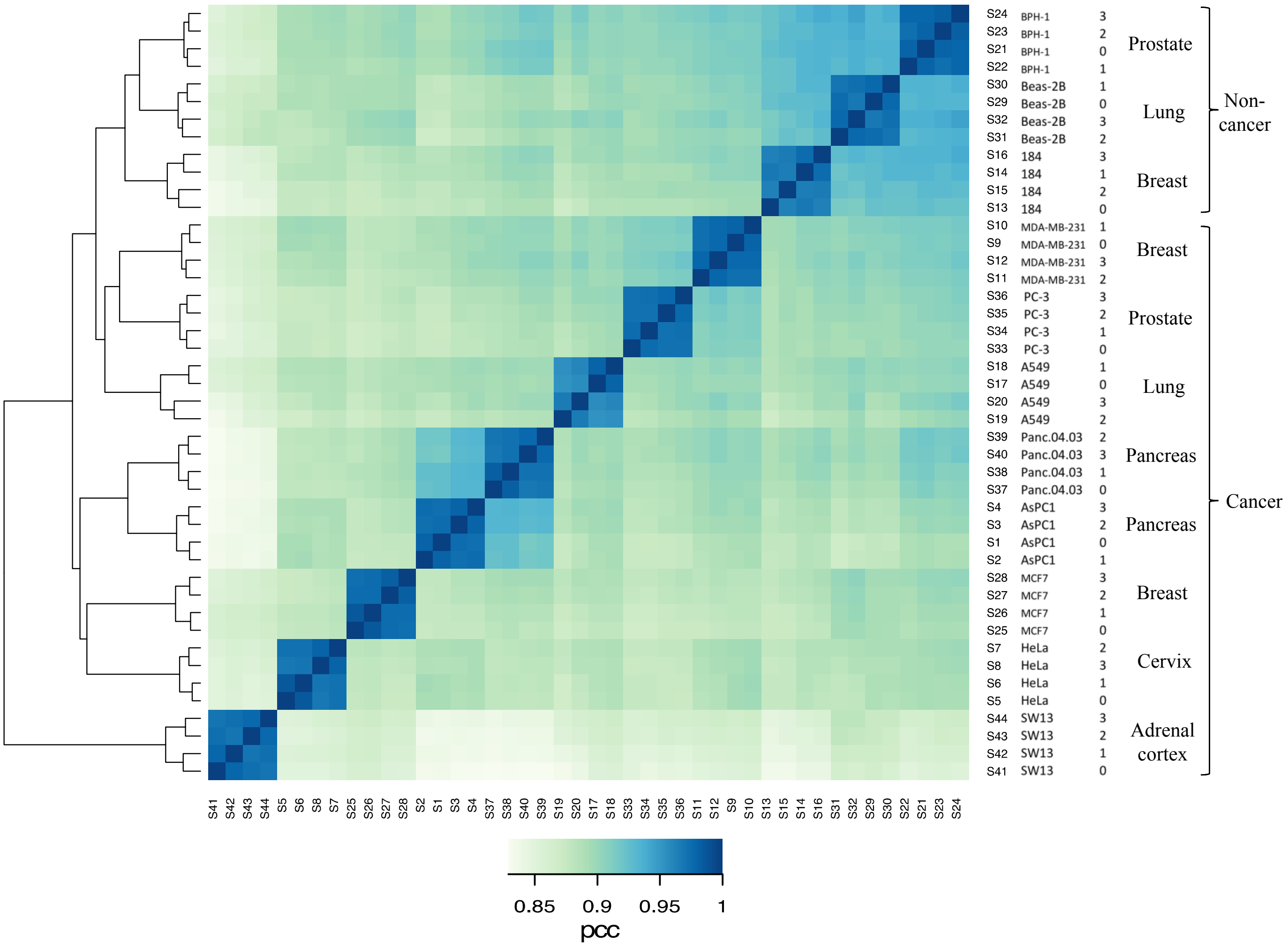

Supplementary Figure K. Correlation Analysis of RNAseq data. Heatmap shows the Pearson Correlation Coefficient (PCC) between the samples calculated from the normalized counts by DESeq (R program). The top left bar shows the PCC scale. The 4 treatments for each cell line were: 0: siCtrl; 1: siCtrl + IFNγ; 2: siSUZ12; and 3: siSUZ12 & IFNγ. The original tissues of the 11 cell lines are indicated to the right: breast (184, non-cancer; MCF7 & MDA-MB-231, cancer), lung (Beas-2B, non-cancer; A549, cancer), prostate (BPH-1, non-cancer; PC-3, cancer), and (Panc.04.03 & AsPC1, cancer), cervix (HeLa, cancer) and adrenal cortex (SW-13, cancer). The clustering shows that samples from same cell line are of the highest similarity, as expected. The clustering also aligned non-cancer cell lines together.

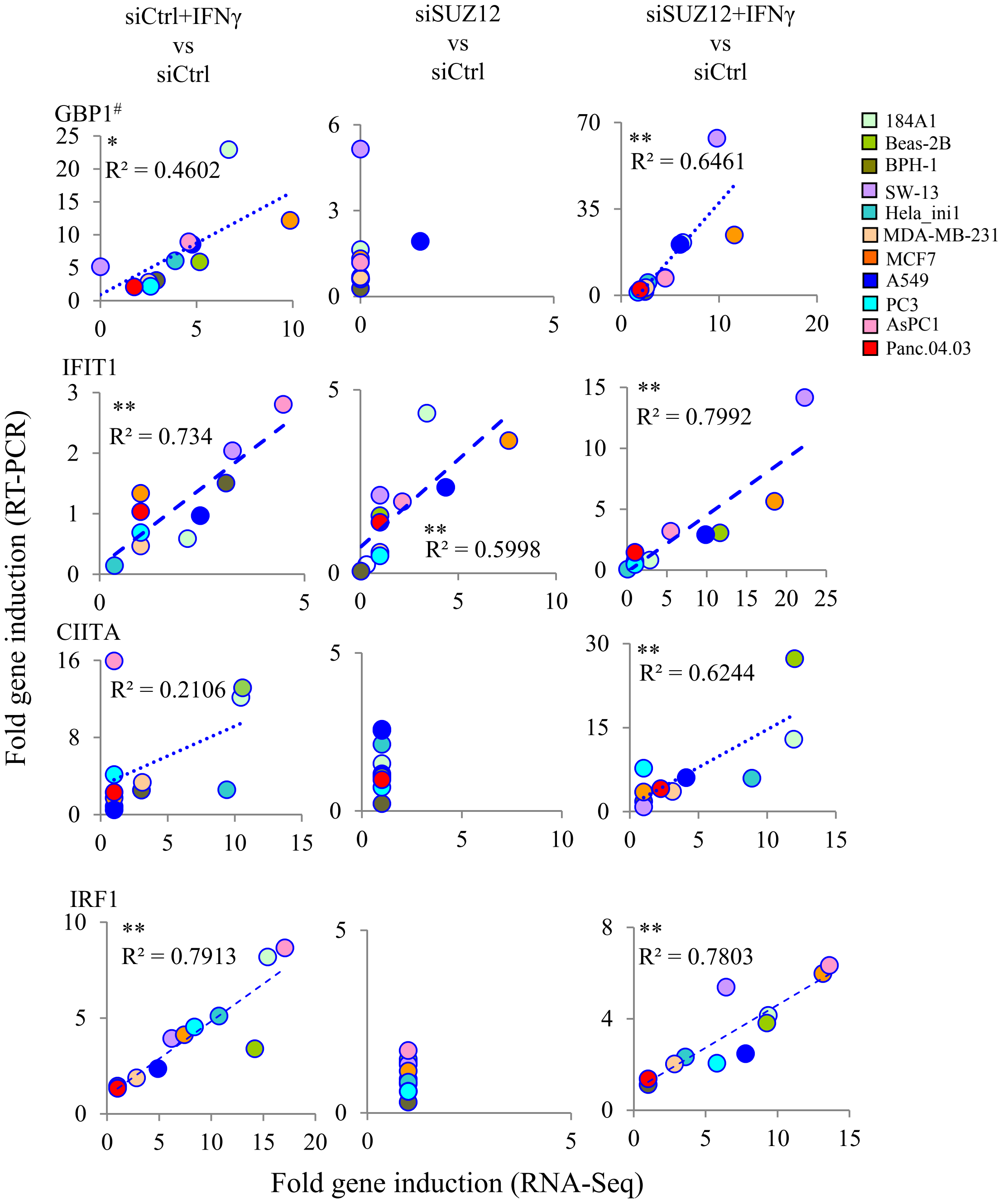

Supplementary Figure L. RNA-seq and RT-PCR data correlate across multiple cell lines. Linear regression analysis was performed to study the agreement between RT-PCR (Supp Fig B) and RNA-seq fold change data for *CIITA*, *GBP1*, *IFIT1*, and *IRF1* across the 11 cell lines assessed; each point on the graph represents a cell line. Separate regression analyses were performed for IFNγ alone, siSUZ12 alone or IFNγ +siSUZ12 compared to controls (siCtrl-IFNγ). P values (*<0.05; **<0.005) were calculated from the square root of the correlation coefficient using GraphPad software.

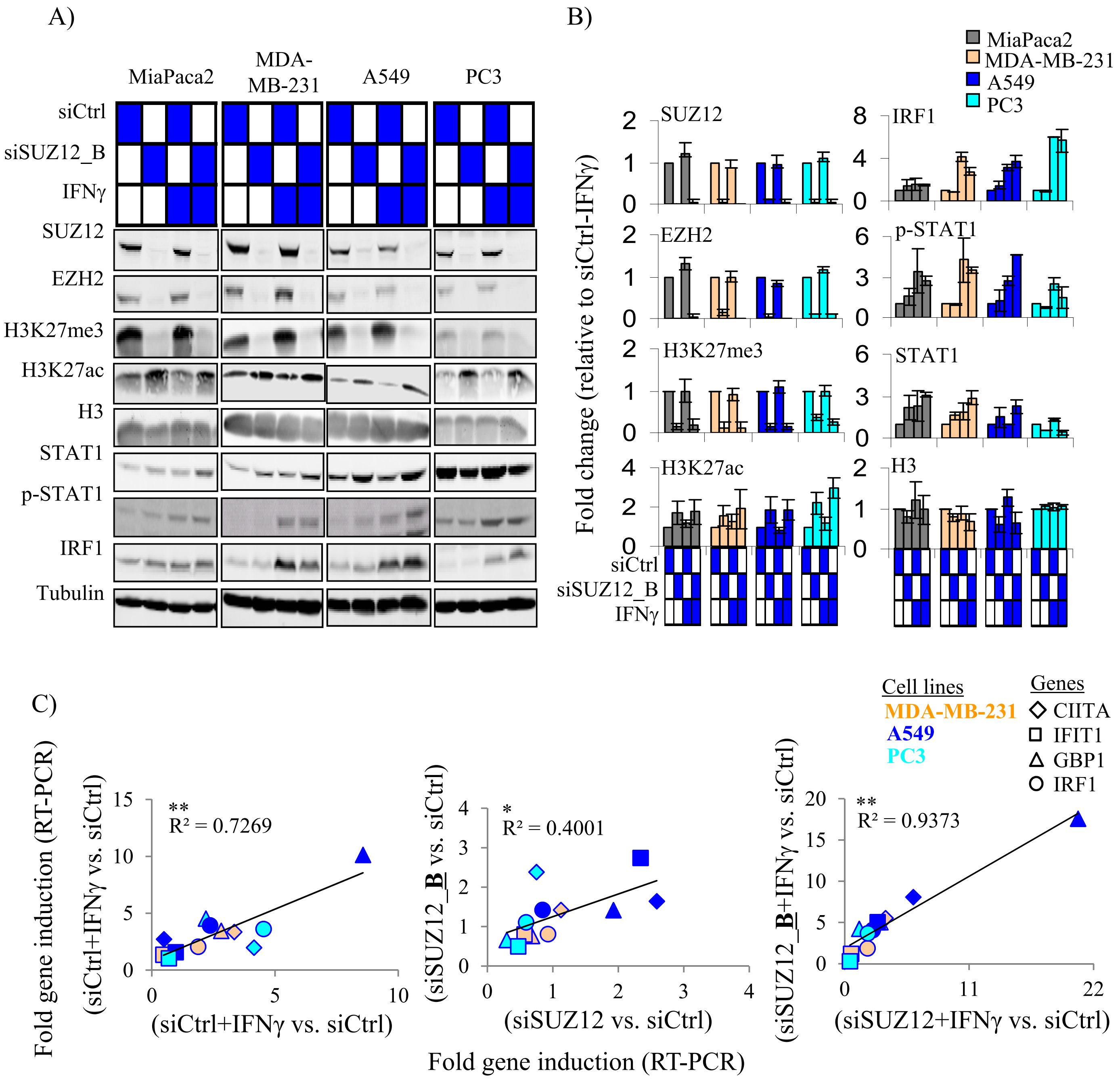

Supplementary Figure M. On-target effects of siSUZ12 on ISG responsiveness. The siSUZ12 reagent used in RNAseq was compared to a second reagent, siSUZ12_B. **(A)** Western blot analyses of the indicated factors or H3 modifications were performed using lysates from the indicated 4 cell lines transfected with siCtrl or siSUZ12 and left untreated or treated with IFNγ for 6 hrs. **(B)** Protein expression was quantified relative to Actin and changes in protein levels were expressed as fold above the value in cells treated with siCtrl and no IFNγ. Values are mean (n=2) +/- range. **(C)** Regression analysis comparing the indicated siRNAs. P values (*<0.05; **<0.005) were calculated from the square root of the correlation coefficient using GraphPad software.

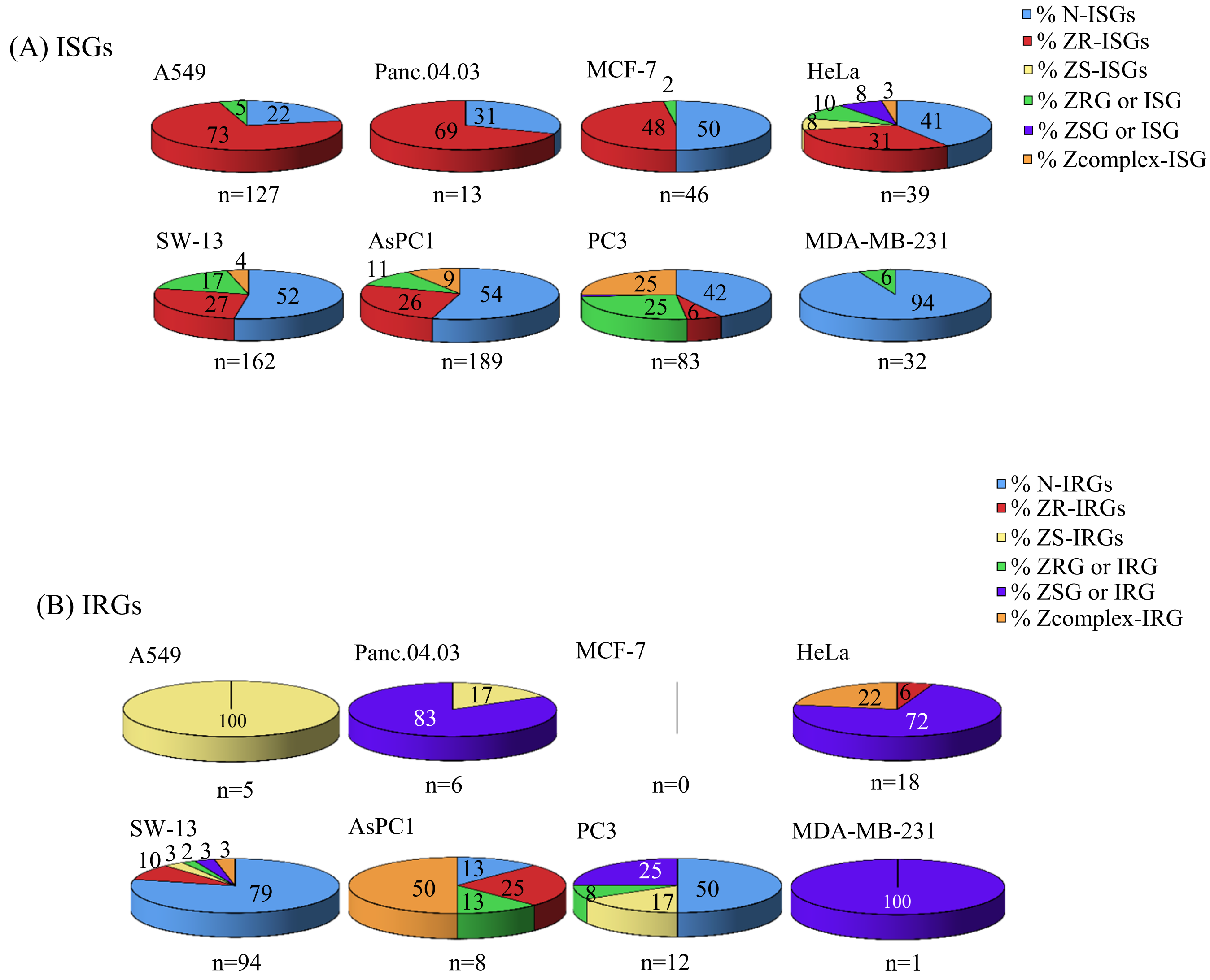

Supplementary Figure N. PRC2 regulates many ISGs but not IRGs. Pie graphs show the percentage of distinct classes of **(A)** ISGs and **(B)** IRGs in different cancer cell lines. Gene classification is based on the response to siSUZ12 and/or IFNγ (details in Supp Table 2).

Gene classes:

*1.N-ISG, **N**ot affected by SUZ12, but an **I**FNγ **S**timulated **G**ene (**ISG**)*

*2.ZR-ISG, SU**Z**12-**R**epressed **ISG***

*3.ZS-ISG, SU**Z**12-**S**timulated **ISG***

*4.ZRG or ISG, SU**Z**12-**R**epressed **G**ene OR **ISG***

*5.ZSG or ISG, SU**Z**12-**S**timulated **G**ene OR **ISG***

*6.Zcomplex-ISG, **ISG**, and while siSU**Z**12 alone has no effect, it counteracts the stimulating effect of IFNγ, thus classified as **complex***

*7.N-IRG, **N**ot affected by SUZ12, but an **I**FNγ-**R**epressed **G**ene (**IRG**)*

*8.ZR-IRG, SU**Z**12-**R**epressed **IRG***

*9.ZS-IRG, SU**Z**12-**S**timulated **IRG***

*10.ZRG or IRG, SU**Z**12-**R**epressed **G**ene OR **IRG***

*11.ZSG or IRG, SU**Z**12-**S**timulated **G**ene OR **IRG***

*12.Zcomplex-IRG, **IRG**. siSU**Z**12 has no effect on gene expression, but counteracts the repressive effect of IFNγ, thus classified as **complex***

More detailed gene description is listed in Supp Table 1

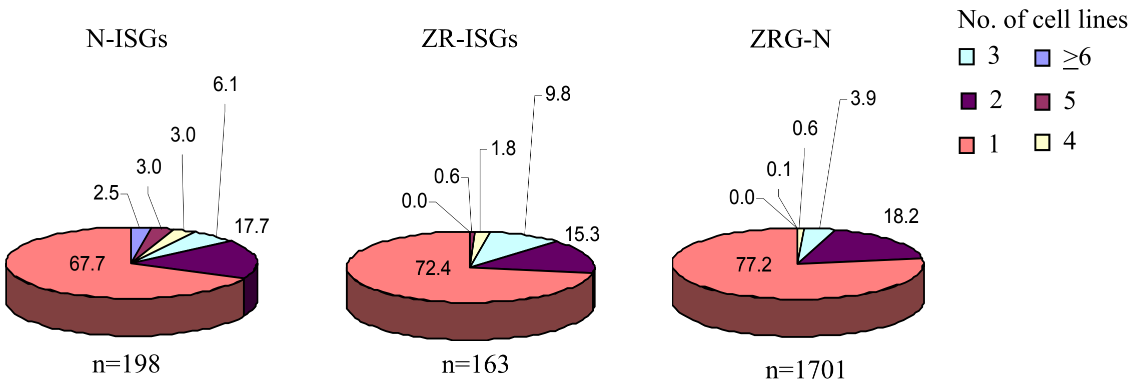

Supplementary Figure O. IFNγ and/or siSUZ12 effects on ISGs are largely cell line dependent. Pie graphs show the frequency with which altered genes ($q \geq 0.9$) of the indicated class were altered across the 11 cell lines tested.

Gene classes:

*1.N-ISG, **N**ot affected by SUZ12, but an **I**FNγ **S**timulated **G**ene (**ISG**)*

*2.ZR-ISG, SU**Z**12-**R**epressed **ISG***

*3.ZRG-N, SU**Z**12 **R**epressed **G**ene, but **N**ot affected by IFNγ*

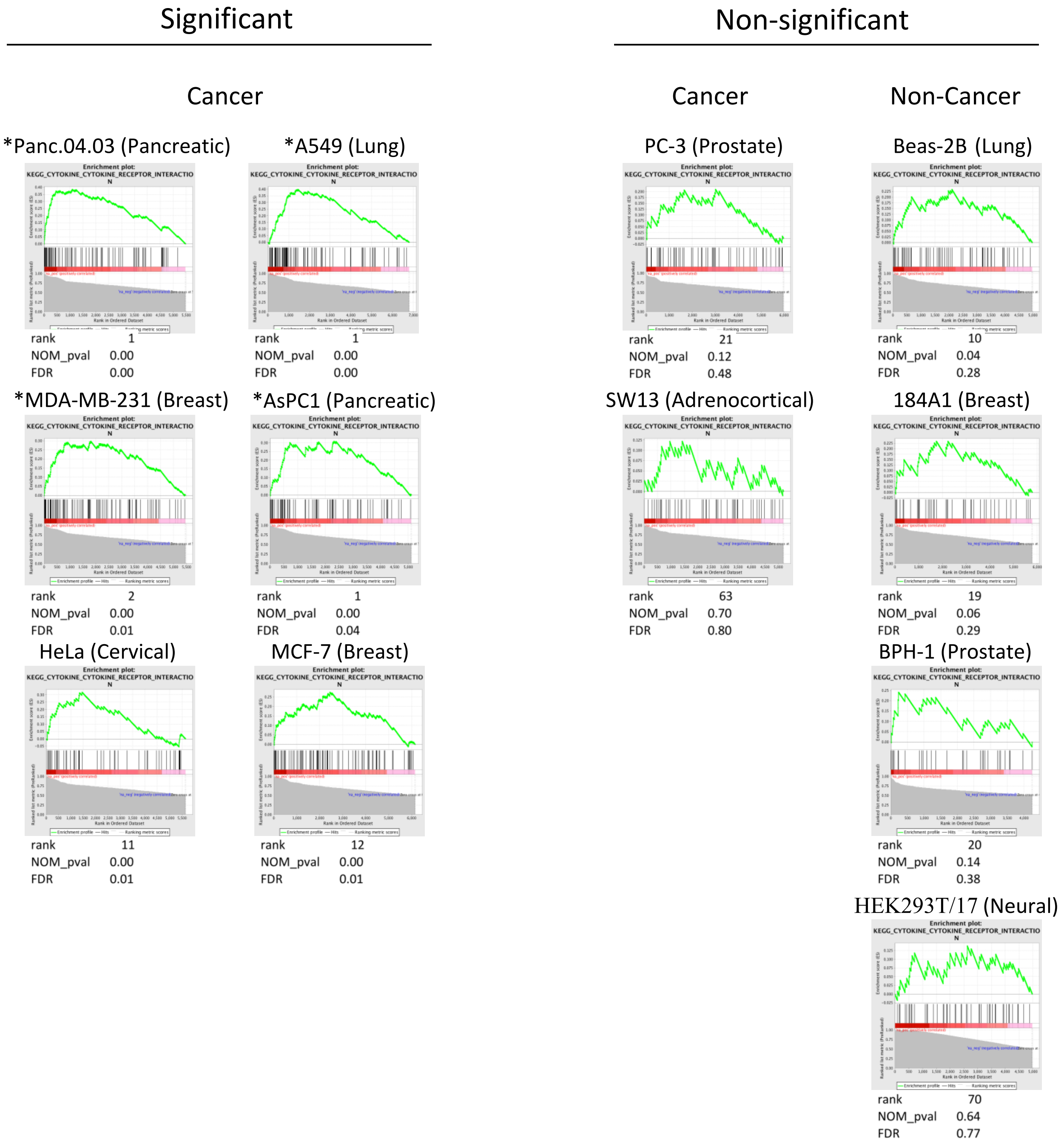

Supplementary Figure P. siSUZ12 induced genes are enriched in CCRI pathway components in multiple cancer cell lines. GSEA enrichment plots are shown for the 8 cancer cell lines and 3 non-cancer derived lines analyzed here by RNAseq, and for the non-cancer-derived HEK293T/17 cell line RNAseq data obtained from the literature. Significant in GSEA: FDR < 0.05 and NOMpval < 0.01. The top portion of each plot (green line) shows the running Enrichment Score (ES) for the CCRI gene set as the analysis walks down the input ranked gene list. The middle portion (vertical black bars) shows where each member of the CCRI gene set appears in the ranked gene list. The bottom portion (gray) shows the value of the ranking metric, which is the probability of differential expression by siSUZ12 *vs*. control. The three parameters below the graph indicate the Rank of the CCRI gene set on the top 20 reported enriched pathways, the nominal P value (NOM_pval) which estimates the statistical significance of enrichment, and the false discovery rate (FDR). * indicates the 4 cancer cell lines where CCRI was the top or second ranked gene set.

A)

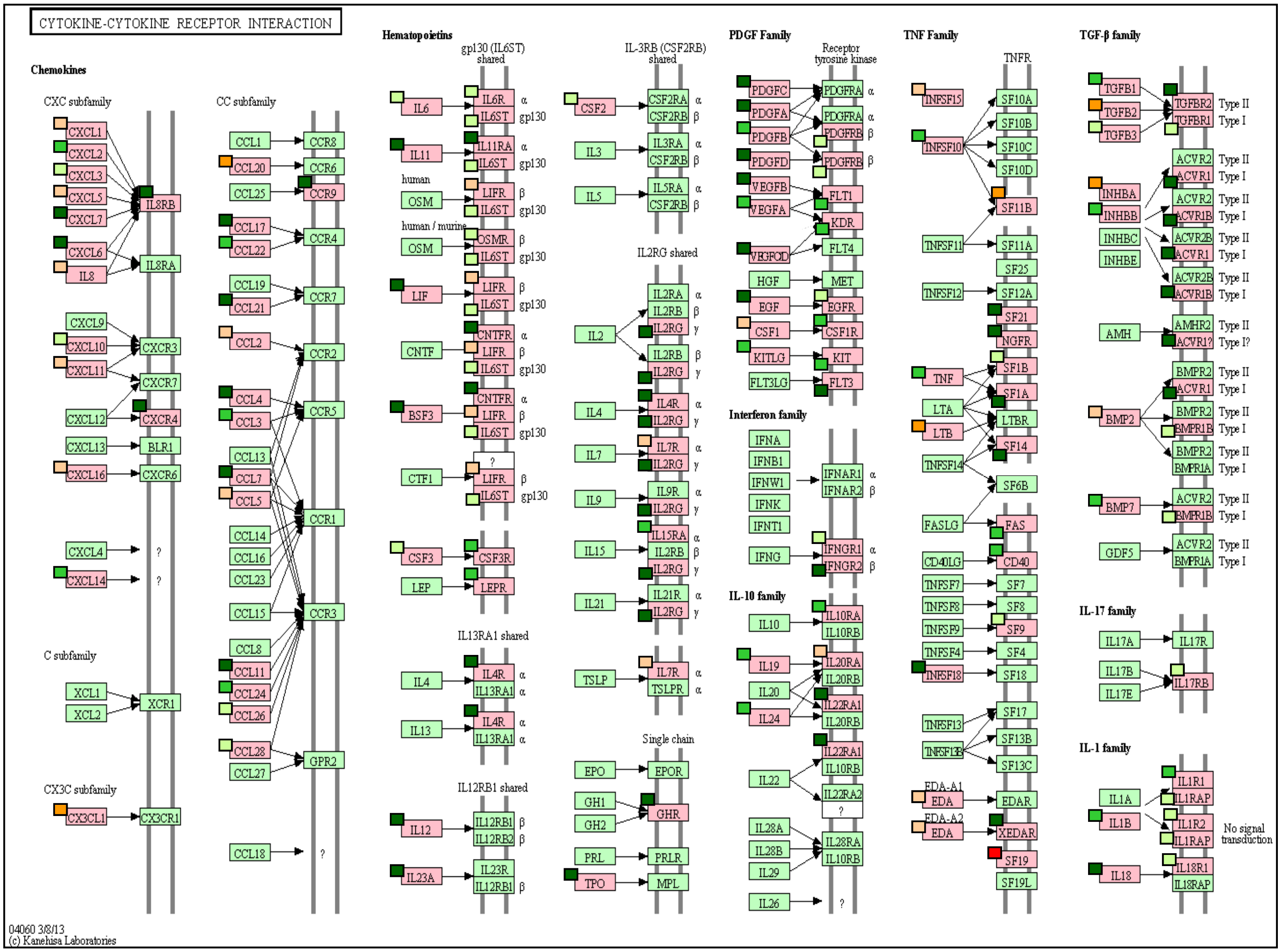

B)

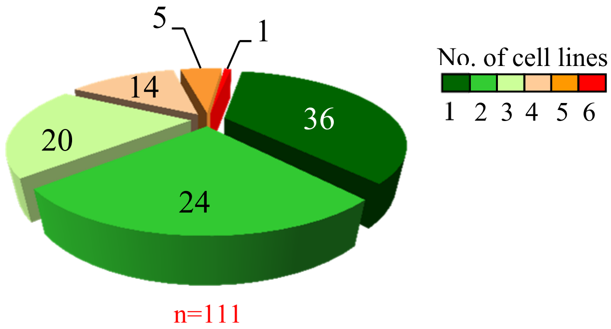

Supplementary Figure Q. PRC2 repressed cytokine-cytokine receptor genes. (A) KEGG pathway mapping of cytokine-cytokine receptor interaction (CCRI) pathway. Of the total of 249 CCRI genes 138 were unaffected (green rectangles) while 111 genes were repressed by PRC2 (Pink rectangles, q ≥ 0.9). The square to the left of each gene indicates the number of cell lines in which the gene was repressed (color key bottom right). (B) Pie graph summarizes the % of PRC2 repressed CCRI genes in ≥ 1 cell lines.

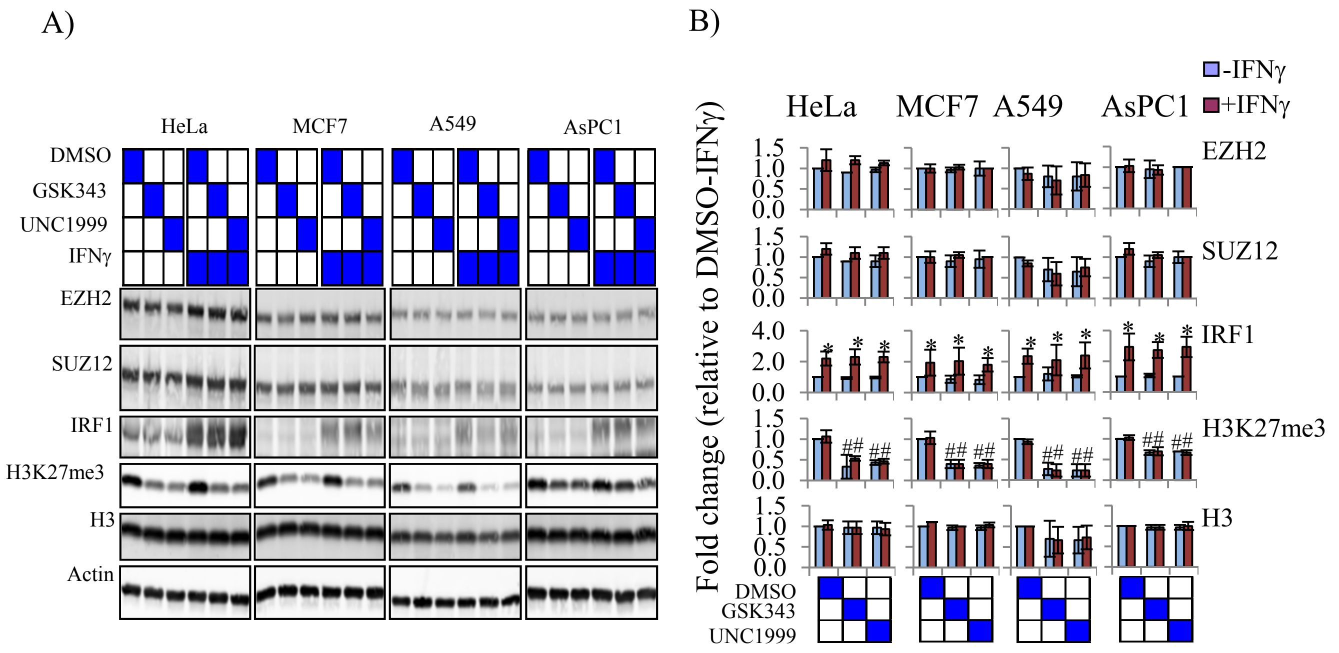

Supplementary Figure R. EZH2 inhibitors reduce PRC2 activity in multiple cancer cell lines. (A) Western blot analyses of the indicated factors, H3K27me3 and H3 were performed using lysates from HeLa, MCF7, A549 and AsPC1 cells treated with DMSO, GSK343 or UNC1999 ± IFNγ for 6 hrs. Treatments are indicated in blue above the blots. **(B)** Graphs show the quantification of Western blots in (A) using Actin as loading control. Changes in protein levels were expressed as fold above the control (DMSO/no IFNγ). Values are mean (n=3) +/- SD. *: Significant ($p<0.05$) induction by IFNγ compared to the matching no IFNγ group. #: Significant ($p<0.05$) reduction by drug compared to DMSO control.

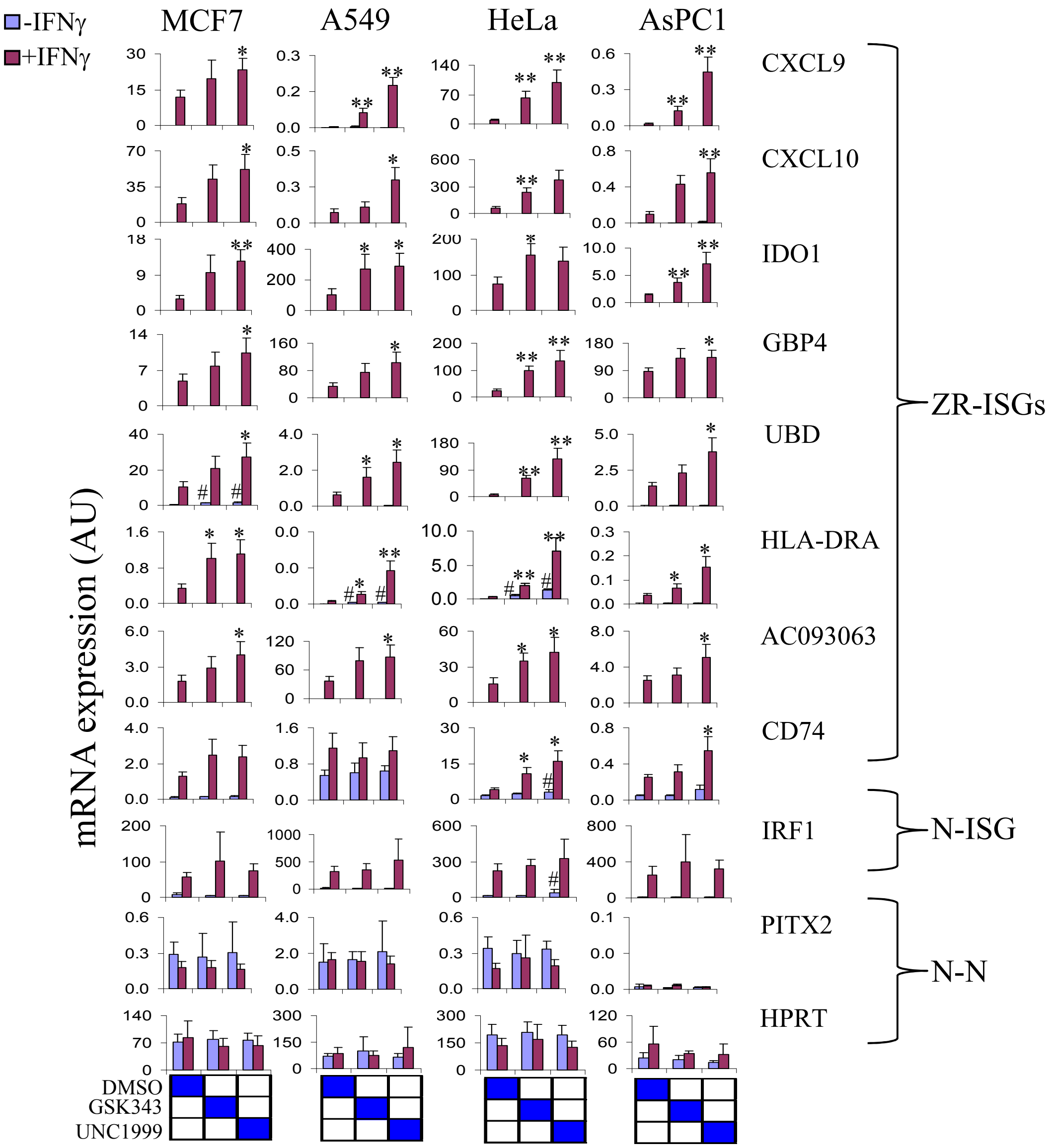

Supplementary Figure S. EZH2 inhibitors augment IFNγ responsiveness of ZR-ISGs in multiple cancer cell lines. Real-time PCR analysis of ISGs identified by RNAseq as SUZ12 repressed (ZR-ISGs), unaffected by SUZ12 (N-ISG; IRF1) and the non-ISGs PITX2 and HPRT in MCF7, A549, HeLa and AsPC1 cells treated with DMSO, GSK343, or UNC1999 ± IFNγ for 6 hrs. Treatments are indicated in blue below the graphs. Gene expression was normalized to Tubulin. Values are mean (n=3) +/- SD. * $p<0.05$ and ** $p<0.01$ (Student's t-test) indicate significant increase of IFNγ responsiveness by the drug compared to DMSO+IFNγ control. # $p<0.05$ indicate significant drug-induced increase in basal expression compared to DMSO control.

Gene classes:

*1.ZR-ISG, SU**Z**12-**R**epressed **ISG***

*2.N-ISG, **N**ot affected by SUZ12, but an **I**FNγ **S**timulated **G**ene (**ISG**)*

3.N-N, Not affected by SUZ12 and/or IFNγ

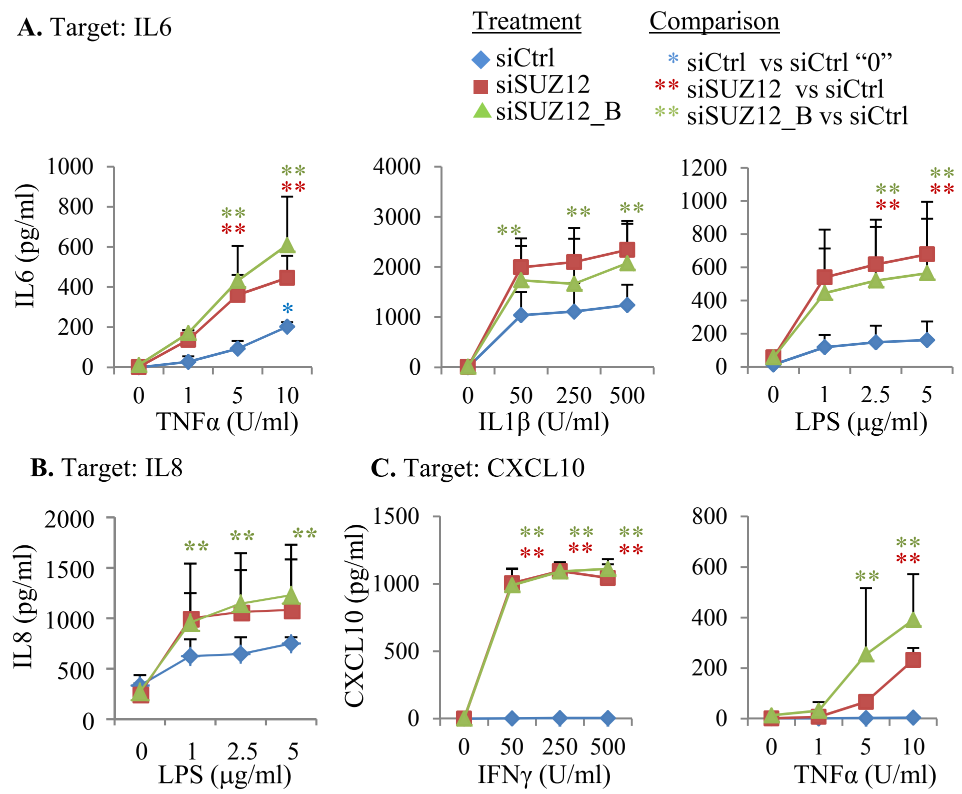

Supplementary Figure T: SUZ12 depletion boosts multiple immune pathways. A549 cells treated with siCtrl or two independent siRNAs for SUZ12 (siSUZ12, siSUZ12_B) were stimulated with the indicated concentrations of TNFα, IFNγ, IL1β, or LPS for 24 h and ELISA was performed for secreted IL6 **(A)**, IL8 **(B)** and CXCL10 **(C)**. Asterisks indicate significant effects ($P < 0.05$; $n = 3$; ANOVA followed by Fisher test) according to the indicated comparisons.
