# Supplementary material for: Cancer Cells Hijack PRC2 to Modify Multiple Cytokine Pathways
Source: PLoS One. 2015 Jun 1;10(6):e0126466. doi: 10.1371/journal.pone.0126466 (PMC4450877; doi:10.1371/journal.pone.0126466)
Supplement: S1 Text — Includes Supplementary Results (more details on the microarray and RT-PCR data in Fig 1 and Supp Figs A-D, as well as a detailed description of RNAseq data validation), and Supplementary Methods. (DOCX) [file pone.0126466.s006.docx]

**S1 Text: SUPPLEMENTARY MATERIALS**

**SUPPLEMENTARY RESULTS**

**Genome-wide antagonism between BRG1 and PRC2 at IFNγ targets**

This section provides a more detailed analysis of the microarray and RT-PCR data in **Fig1** and **Supp Figs A-B**.

Focusing first on basal effects, BRG1 and siSUZ12 altered 465 genes, or 2% of all loci, affecting genes linked developmental regulation, signal transduction and cell migration (**Fig 1A**). Basal expression of only 12 known IFNγ targets were affected, 11 induced and one repressed (**Fig 1A**). As described below, we detected 109 ISGs, thus SWI/SNF or PRC2 affect basal expression of only a few (~10%) ISGs.

In total, BRG1 stimulated (BrS) basal expression of 221 genes, of which 22% were also induced by siSUZ12 (SUZ12-repressed genes; BrS/ZRG), 2% were down-regulated by siSUZ12 (SUZ12-stimulated genes, BrS/ZSG) and most (76%) were unaffected by siSUZ12 (BrSG) (**Fig 1A**). siSUZ12 up-regulated 168 genes (SUZ12-repressed, ZR), of which 29% were also BRG1-stimulated (BrS/ZRG), 0.5% were repressed by BRG1 (BrR/ZRG), and 71% were unaffected by BRG1 (ZRG). Of all up-regulated genes, a modest 14% (48/342) were both BRG1 and PRC2 targets.

In total, BRG1 repressed (BrR) basal expression of 77 genes, of which 26% were also down-regulated by siSUZ12 (BrR/ZSG), <1% were up-regulated by siSUZ12 (BrR/ZRG) and most (73%) were unaffected by siSUZ12 (BrRG) (**Fig 1A**). siSUZ12 down-regulated (ZS) 72 genes, of which 28% were also BRG1-repressed (BrR/ZSG), 7% were stimulated by BRG1 (BrS/ZSG), and 65% were unaffected by BRG1 (ZSG). Of all down-regulated genes only 16% were both BRG1 and siSUZ12 targets.

In summary, SWI/SNF and/or PRC2 regulate the basal expression of a small fraction of all genes, and common targets are regulated in opposite directions, but co-regulation is modest (≤ ~1/6^th^).

Next, we assessed how BRG1 or siSUZ12 affect IFNγ responsiveness. In total, BRG1 rescued or enhanced IFNγ-stimulation of 71 ISGs. Notably, siSUZ12 rescued almost half these SWI/SNF targets in BRG1-deficient cells (BrS/ZR-ISGs, 45%), with the remainder rescued by BRG1 but not siSUZ12 (BrS-ISGs) (**Fig 1B**). In total, siSUZ12 rescued IFNγ-induction of 56 genes, of which 57% were BRG1-dependent (BrS/ZR-ISGs), and the remainder were SUZ12-repressed but BRG1-independent (ZR-ISGs). Like all genes (**Fig 1A**), BRG1 and SUZ12 had antagonistic effects on shared ISGs (**Fig 1B**). Of all rescued ISGs, 34% (32/95) were both BRG1 and siSUZ12 targets, 2.5-fold higher than the 14% of all genes. Of all 109 ISGs, a striking 87% required BRG1 and/or siSUZ12 to be induced at all or maximally by IFNγ (**Fig 1B**), contrasting starkly with the 2% of all genes affected by these regulators (**Fig 1A**).

We validated the array data using RT-PCR analysis. First, we assessed BRG1-dependency at 52 well known ISGs. SW-13 cells were transduced with adenovirus expressing BRG1 (Ad-BRG1) and left untreated or IFNγ-stimulated for 6 hours and ISG expression tested by RT-PCR. IFNγ-responsiveness was mostly BRG1-dependent (**Fig A in S1 File**). We further assessed the effect of siSUZ12 on 7 BRG1-dependent (*GBP1, GBP2, IFI6, IFITM1, IFITM3, IFIT1, IFIT5*), 2 BRG1-independent (*PSMB9, STAT1*) genes and 2 control genes (*HPRT, PITX2*). SUZ12 knockdown (**Fig B, Panel A in S1 File**) enhanced IFNγ-responsiveness at 5/7 BRG1-dependent genes, while BRG1-independent ISGs or control genes were unaffected (**Fig B, Panel B in S1 File**). These data confirm an extensive, antagonistic role for SWI/SNF and PRC2 in IFNγ responsiveness.

**Validation of RNA-seq Data**

Forty four assays were run to assess the effect of SUZ12 knockdown on basal or IFNγ-induced gene expression (4 conditions x 11 lines). Numerous tests confirmed that the data were high quality. Both principal component analysis (PCA) and correlation analysis showed that the 4 datasets (+/- siSUZ12, +/- IFNγ) for each line were more related to each other than to those from other lines (**Fig J & K in S1 File**). Also, we used a six-component pattern analysis to define genes with robust differential expression (see Methods), and SUZ12-repressed ISGs (ZR-ISGs) had the fourth highest median number of genes/cell line (22) ranking behind unaffected genes (N-N, 8146), SUZ12-repressed genes unaffected by IFNγ (ZRG-N, 218), and ISGs unaffected by SUZ12 (N-ISG, 30) (**S1 Table**). Unusual gene classes (e.g. SUZ12 stimulated IFNγ repressed genes, ZS-IRG) were either absent or present at a median of ≤6 genes/cell line (see Methods, & **S1 Table**). Four ISGs were validated across all 11 lines and RNA-seq and RT-PCR data correlated (**Fig L in S1 File).** These four ISGs were also validated in four cell lines using a different SUZ12 siRNA, and neither siRNA affected STAT1, pSTAT1, or IRF1 protein (**Fig M in S1 File**). As described in the main text we validated additional targets, both by RT-PCR and ELISA (**S2 Table**), and pharmaceutical EZH2 inhibitors further validated PRC2 targets at the RNA and protein level (see main text, **Fig 7**, **Fig R-T in S1 File**).

**SUPPLEMENTARY METHODS**

*ChIP on chip data analysis*

To set a maxM/P cutoff value, we measured H3K27me3 levels at 30 promoters using ChIP-qPCR and used this dataset as a reference to test the specificity, sensitively and accuracy (equations 1-3) of the ChIP-chip data and define the most accurate maxM/P cutoff value.

We obtained the highest sensitivity, specificity and accuracy of ChIP-chip detection using maxM/P values ranging from 0.3 to 0.7. To minimize false positives 0.7 was selected as the cutoff value and the accuracy 80%. Then we aligned ChIP-chip peaks to the chromosome locations located at +/-1kb around the TSS of all genes with a resolution of 100 bp. Next, we calculated the average H3K27me3 levels per gene by calculating the mean value of H3K27me3 intensities across all bins within the +/-1kb region around the TSS of each gene and normalized it to the average probe density across all bins. Then we calculated the average H3K27me3 levels per gene for a specific group of genes. We also calculated the distribution of H3K27me3 signature around the TSS of genes. To this end, we calculated the average H3K27me3 levels per bin across all genes of a specific category and normalized values to the average probe density per bin. We repeated calculations for all bins within the studied 2kb region and drew the average intensity per bin as a function of the bin distance from the TSS.

*RNA-seq Differential Expression Analysis*

After counts were assembled, they were normalized using R package DESeq [1] to limit possible batch effects. Then, sample clustering and Principle Components Analysis (PCA) were applied to assess data quality. We calculated the Euclidean distances from sample to sample and visualized the clustered distance matrix in a heatmap with dendrograms to view sample relationships. PCA was applied to the top 500 genes with greatest variances between samples. The first two components were plotted against each other in a scatter plot. All calculations and visualizations were performed in R.

Differential expression was assessed using the R package NOISeq [2]. Because we had a single biological replicate for each of the 44 samples, we applied a rigid, logic driven criteria to select genes with robust differential expression patterns that were consistent across six different comparisons, and discarded genes with illogical patterns:

1. IFNγ *vs.* Ctrl

2. siSUZ12 *vs.* Ctrl

3. siSUZ12IFNγ *vs.* Ctrl

4. siSUZ12IFNγ *vs.* IFNγ

5. siSUZ12IFNγ *vs.* siSUZ12

6. IFNγ *vs.* siSUZ12

Each position in the six letter differential expression (DE) pattern was given one of three letters: “U” meaning “Up” (> 1.5 fold increased expression); “D” meaning “Down” (>1.5 fold decreased expression), and “N” meaning “No change” (<1.5 fold change). For example, the code "**NUUUND**" indicates:

1. No difference between IFNγ and Ctrl treatment groups (**N**)

2. Higher expression in siSUZ12 treatment group compared to Ctrl group (**U**)

3. Higher expression in siSUZ12 + IFNγ treated group compared to Ctrl group (**U**)

4. Higher expression in the siSUZ12+IFNγ treatment group compared to IFNγ only group (**U**)

5. No difference in expression between siSUZ12+IFNγ and siSUZ12 treatment groups (**N**)

6. Lower gene expression in IFNγ-treated group compared to siSUZ12-treated group (**D**)

The DE pattern is a strict assessment because parameters must be internally consistent or the gene designation will be illogical. The total number of theoretical DE patterns is 729 (3^6^), but most are illogical, e.g. NNUNNN, but in practice some of the latter type are present because of values around the 1.5x threshold and/or data noise. Of the 107976 transcripts we detected across all 11 lines (average 9816 per cell type) they fitted 120 of the 729 theoretical DE patterns, of which 36 were logical, and accounted for the vast majority of transcripts (93792 /107976 or 86.9%); moreover, and also as expected, most transcripts (88581/107976 or 82.0%) were unchanged (UUUUUU) in any condition (**Table S2**). Among the transcripts that changed with one or more treatment the most abundant classes were expected, including genes repressed by SUZ12 but unaffected by IFNγ (ZRG-N), genes unaffected by SUZ12 but stimulated by INFγ (N-ISG), and ISGs that were repressed by SUZ12 (ZR-ISG). Altogether, these findings argue that the RNAseq data is reliable, and additional analyses and validation further supported this conclusion (see above and Results).

*ChIP-Seq data analysis*

ChIP-seq data for H3K27me3 and H3K4me3 in A549 cells were downloaded from ENCODE/Broad institute histone analyses (UCSC Accessions: wgEncodeEH003120 and wgEncodeEH003065). We mapped BAM data to -5 kb to + 5 kb relative to transcription start sites (TSS) binned by 100 bp windows, then applied unsupervised K-mean clustering based on K27me3 and K4me3 ChIP-seq signals, which generated 10 gene clusters. We ordered the 10 clusters by the average log_2_Fold changes in gene expression after siSUZ12 treatment for each cluster, and within each cluster, sorted genes into three bins according to low, medium, or high basal expression. The clustered signals were visualized as a heatmap aligned with dot plots of the expression log_2_Fold change after siSUZ12 treatment for each gene. Subsequently, we evaluated, within the whole mapped gene set, the enrichment of H3K27me3 signals in four different gene groups: all siSUZ12 induced genes (ssGenes), all genes not affected by siSUZ12 (nssGenes), CCRI genes induced by siSUZ12 (ssCCRI), CCRI genes unaffected by siSUZ12 (nssCCRI). For the evaluation, the average H3K27me3 signal at each window within -5 kb to + 5 kb were compared between each of the groups and a random gene set sampled from the whole gene set in size equal to the median sample size of the 4 groups. Each pair of comparisons was run 1000 times with an individual draw of the random set. For each run, the significant difference between a sample set and a random set was tested by a non-parametric Wilcox paired test with the alternative hypothesis as sample set > random set and confidence level = 0.99. Then, the enrichment p value was determined by the proportion of rejections against the above alternative hypothesis out of the 1000 runs. Finally, to determine whether the genes induced by siSUZ12 and with significant H3K27me3 repression features exhibit a bivalent H3K27me3/H3K4me3 signature, we further evaluated the enrichment of H4K4me3 signals for these four groups of genes in a subset of genes with significant repression signatures, *i.e.* the genes in cluster 1-4. The evaluation followed the same approach as above except within -3 kb to + 3 kb relative to the TSS since, in general and as showed in our clustered heatmap, bivalent markers are H3K27me3 dominated and with weaker and narrower H3K4me3 markers. Data in this analysis was processed, computed and visualized by customized programs written in R, as well as customer modified functions of the Repitools package [3]**.**

**SUPPLEMENTARY REFERENCES**

1. Anders S, Huber W. Differential expression analysis for sequence count data. Genome Biol. 2010;11: R106. doi:10.1186/gb-2010-11-10-r106

2. Tarazona S, García-Alcalde F, Dopazo J, Ferrer A, Conesa A. Differential expression in RNA-seq: a matter of depth. Genome Res. 2011;21: 2213–2223. doi:10.1101/gr.124321.111

3. Statham AL, Strbenac D, Coolen MW, Stirzaker C, Clark SJ, Robinson MD. Repitools: an R package for the analysis of enrichment-based epigenomic data. Bioinforma Oxf Engl. 2010;26: 1662–1663. doi:10.1093/bioinformatics/btq247

**SUPPLEMENTARY FIGURE LEGENDS**

**Fig A. Multiple ISGs are BRG1-dependent.** **(A)** Real-time PCR analysis of 55 ISGs in SW-13 cells transduced with Ad-GFP or Ad-BRG1 for 24 hours then left untreated or treated with IFNγ for 6 hrs. Gene expression was normalized to Tubulin and presented in arbitrary units (AU). Genes with AU of < 2 are considered silent (off). Values are mean (n > 3) +/- S.D. *, †, and $ indicate significantly changed genes (P < 0.05, ANOVA followed by Fisher test) according to the indicated comparisons (key). ISGs are presented according to responsiveness to IFNγ into resistant (Off/off, On/same) or induced genes (Off/on, On/up), and induced genes were further sorted according to BRG1 dependency. **(B)** Pie graph summarizing BRG1 dependency of responsive ISGs (Off/on and On/up).

**Fig B. PRC2-depletion rescues responsiveness of BRG1-dependent ISGs in BRG1-deficient cells. (A)** Representative Western blots of the indicated factors and histone modifications. Analyses were performed using lysates of SW-13 cells treated with siCtrl or siSUZ12 alone or in combination with siBRM. siRNA treatments are indicated by blue squares. Data were reproduced at least twice and protein levels quantified relative to Tubulin. Asterisks indicate significant changes compared to siCtrl group (p<0.05, ANOVA followed by Fisher test). **(B)** Real-time PCR analysis of the indicated genes in SW-13 cells transfected with siCtrl or siSUZ12 alone or in combination with siBRM and left untreated or exposed to IFNγ for 6 hrs, as indicated in blue in the table. Gene expression was normalized to Tubulin and presented in arbitrary units (AU). Values are mean (n > 3) +/- S.D. *, **, †, ‡ indicate significant changes (p<0.05, ANOVA followed by Fisher test) according to the indicated comparison (key).

**Fig C. Rescue of ISGs responsiveness by siSUZ12 is not due to an off target effect.** Real-time PCR analysis of the indicated genes in SW-13 cells transfected with siCtrl or siSUZ12_B and left untreated or exposed to IFNγ for 6 hrs. Gene expression was normalized to Tubulin and presented in arbitrary units (AU). Values are mean (n > 3) +/- S.D. #, * and † indicate significant changes (P < 0.05, ANOVA followed by Fisher test) according to the indicated comparisons (key).

**Fig D. BRM depletion has minimal effect on the expression levels of SUZ12-repressed ISGs.** Microarrays were performed with RNA from cells exposed to siSUZ12 or siSUZ12 + siBRM and left untreated or exposed to IFNγ. For the vast majority of genes fold induction by IFNγ after SUZ12 knockdown was similar (<1.5 fold difference) whether BRM was depleted or not (blue circles), and very few ISGs were up- (red) or down-regulated (green) ≥1.5 fold.

**Fig E. siSUZ12-rescued ISGs are direct PRC2 targets.** (**A**) ChIP-qPCR on chromatin from SW-13 cells was performed to assess basal H3K27me3 levels at selected SUZ12-dependent and independent ISG promoters. Data are % of input DNA. Values are mean (n ≥ 3) +/- SD. * indicates ISG promoters with significant basal H3K27me3 compared to IRF1 promoter (dashed line; p<0.05, ANOVA followed by Fisher test). (**B**) Average H3K27me3 levels at SUZ12-rescued ISGs or positive control gene promoters (shown in A) are significantly higher (* p<0.0005, Student’s t test) than H3K27me3 levels at SUZ12-independent ISGs.

*Gene classes:*

1. *BrS/ZR-ISG,* ***BR****G1-****S****timulated and SU****Z****12-****R****epressed* ***I****FNγ* ***S****timulated* ***G****ene (****ISG)***
2. *ZR-ISG, SU****Z****12-****R****epressed* ***ISG***
3. *BrS-ISGs,* ***BR****G1-****s****timulated* ***ISG***
4. *N-ISG,* ***N****ot affected by SUZ12, but an* ***ISG***

**Fig F. Promoter H3K27me3 anti-correlates with gene expression**. ChIP-chip was performed using chromatin from SW-13 cells and a genome-wide promoter array. **(A)** ChIP-chip signal intensity in 100 bp bins +/-5 kb of the TSS of All genes. **(B)** Histogram of the percentage of All gene promoters positive or negative for H3K27me3. **(C)** ChIP-chip signal intensity as in (A) but grouped according to basal expression. **(D)** Histogram of the percentage of H3K27me3 positive and negative promoters among basally silent (Off) or active genes expressed at the indicated level.

**Fig G. H3K27me3 is enriched at siSUZ12-induced genes.** **(A)** Color-code for gene classes in (B)-(D). **(B)** Heatmap shows basal H3K27me3 ChIP-chip signal within +/-1 kb of the TSS of 445 differentially expressed genes with annotated TSSs in siSUZ12 or AdBRG1 treated cells presented in Fig 1A. **(C)** ChIP-chip signal intensity per 100 bp bins within +/-1kb of the TSS of each gene class. **(D)** Violin plot shows the average normalized ChIP-chip signal across 100bp bins +/-1 kb of the TSS of each gene class (* P < 0.05; Mann Whitney test). **(E)** Histogram shows the percentage of H3K27me3 positive and negative promoters in each indicated gene class. *: significantly higher % of H3K27me3 positive genes between the indicated groups (P < 0.05, Fisher exact test). Abbreviations: Br: Brg1: Z: SUZ12, S: Stimulated, R: Repressed; G: Gene.

*Gene classes:*

1. *BrS/ZRG,* ***BR****G1-****S****timulated and SU****Z****12-****R****epressed* ***G****ene*
2. *ZRG, SU****Z****12-****R****epressed* ***G****ene*
3. *BrSG,* ***BR****G1-****s****timulated* ***G****ene*
4. *BrR/ZSG,* ***BR****G1-****R****epressed and SU****Z****12-****S****timulated* ***G****ene*
5. *ZSG, SU****Z****12-****S****timulated* ***G****ene*
6. *BrRG,* ***BR****G1-****R****epressed* ***G****ene*
7. *BrS/ZSG,* ***BR****G1-****S****timulated and SU****Z****12-****S****timulated* ***G****ene*
8. *BrR/ZRG,* ***BR****G1-****R****epressed and SU****Z****12-****R****epressed* ***G****ene*

**Fig H. Basal expression of SWI/SNF, PRC2 subunits and H3 modifications in a panel of non-cancer (green) and cancer (red) derived cell lines.** **(A)** Western blots show the basal expression of the indicated proteins. Cells were segregated based on their tissue of origin (non-cancer-derived control cells green, cancer cells red). Protein levels were normalized to Actin. Normalized protein expression levels in cancer cells are presented below the blots as fold above the levels in the non-cancer derived cells of the same tissue origin. **(B)** Regression analysis indicated a correlation between the PRC2 subunits EZH2 and SUZ12, but not other protein pairs.

**Fig I. siSUZ12 inhibits H3K27 tri-methylation in human non-cancer and cancer derived cell lines. (A)** Western blot analyses of the indicated SWI/SNF and PRC2 subunits, IRF1 and total and modified histone H3 were performed using lysates of non-cancer (green) and cancer (red) derived cell lines. Cells were transfected with siCtrl or siSUZ12 and left untreated or stimulated with IFNγ for 6 hrs, as indicated by blue squares below the blots. **(B)** Protein expression was quantified relative to Actin and changes in protein levels were expressed as fold above the siCtrl minus IFNγ control. Values are mean (n=2) +/- range.

**Fig J. Principal Component Analysis (PCA) of RNAseq data.** 44 samples of 11 cell lines and 4 treatments are shown in the 2D plane spanned by their first two principal components based on the top 500 genes with the largest variance**.** The overall effects of covariates are from differences between cancer and non-cancer and from differences between cell lines. No batch effects were detected. The original tissues of the 11 cell lines are: breast (184, non-cancer; MCF7 & MDA-MB-231, cancer), lung (Beas-2B, non-cancer; A549, cancer), prostate (BPH-1, non-cancer; PC-3, cancer), pancreas (Panc.04.03 & AsPC1, cancer), cervix (HeLa, cancer) and adrenal cortex (SW-13, cancer).

**Fig K. Correlation Analysis of RNAseq data.** Heatmap shows the Pearson Correlation Coefficient (PCC) between the samples calculated from the normalized counts by DESeq (R program). The top left bar shows the PCC scale. The 4 treatments for each cell line were: 0: siCtrl; 1: siCtrl + IFNγ; 2: siSUZ12; and 3: siSUZ12 & IFNγ. The tissue of origin of the 11 cell lines are indicated to the right. The clustering shows that samples from same cell line are of the highest similarity, as expected. The clustering also aligned non-cancer cell lines together.

**Fig L. RNA-seq and RT-PCR data correlate across multiple cell lines.** Linear regression analysis was performed to study the agreement between RT-PCR (Fig B) and RNA-seq fold change data for *CIITA*, *GBP1*, *IFIT1*, and *IRF1* across the 11 cell lines assessed; each point on the graph represents a cell line. Separate regression analyses were performed for IFNγ alone, siSUZ12 alone or IFNγ +siSUZ12 compared to controls (siCtrl-IFNγ). P values (*<0.05; **<0.005) were calculated from the square root of the correlation coefficient using GraphPad software.

**Fig M. On-target effects of siSUZ12 on ISG responsiveness.** The siSUZ12 reagent used in RNAseq was compared to a second reagent, siSUZ12_B. **(A)** Western blot analyses of the indicated factors or H3 modifications were performed using lysates from the indicated 4 cell lines transfected with siCtrl or siSUZ12 and left untreated or treated with IFNγ for 6 hrs. **(B)** Protein expression was quantified relative to Actin and changes in protein levels were expressed as fold above the value in cells treated with siCtrl and no IFNγ. Values are mean (n=2) +/- range. **(C)** Regression analysis comparing the indicated siRNAs. P values (*<0.05; **<0.005) were calculated from the square root of the correlation coefficient using GraphPad software.

**Fig N. PRC2 regulates many ISGs but not IRGs.** Pie graphs show the percentage of distinct classes of **(A)** ISGs and **(B)** IRGs in different cancer cell lines. Gene classification is based on the response to siSUZ12 and/or IFNγ (details in Table S2). Abbreviations: N: Not affected by SUZ12; Z: SUZ12, I: Interferon-γ; R: Repressed; S: Stimulated; G: Gene.

*Gene classes:*

1. *N-ISG,* ***N****ot affected by SUZ12, but an* ***I****FNγ* ***S****timulated* ***G****ene (****ISG****)*
2. *ZR-ISG, SU****Z****12-****R****epressed* ***ISG***
3. *ZS-ISG, SU****Z****12-****S****timulated* ***ISG***
4. *ZRG or ISG, SU****Z****12-****R****epressed* ***G****ene OR* ***ISG***
5. *ZSG or ISG, SU****Z****12-****S****timulated* ***G****ene OR* ***ISG***
6. *Zcomplex-ISG,* ***ISG****, and while siSU****Z****12 alone has no effect, it counteracts the stimulating effect of IFNγ, thus classified as* ***complex***
7. *N-IRG,* ***N****ot affected by SUZ12, but an* ***I****FNγ-****R****epressed* ***G****ene (****IRG****)*
8. *ZR-IRG, SU****Z****12-****R****epressed* ***IRG***
9. *ZS-IRG, SU****Z****12-****S****timulated* ***IRG***
10. *ZRG or IRG, SU****Z****12-****R****epressed* ***G****ene OR* ***IRG***
11. *ZSG or IRG, SU****Z****12-****S****timulated* ***G****ene OR* ***IRG***
12. *Zcomplex-IRG,* ***IRG****. siSU****Z****12 has no effect on gene expression, but counteracts the repressive effect of IFNγ, thus classified as* ***complex***

*More detailed gene description is listed in Table S1*

**Fig O. IFNγ and/or siSUZ12 effects on ISGs are largely cell line dependent.** Pie graphs show the frequency with which genes of the indicated class were altered (q ≥ 0.9) across the 11 cell lines tested. ). Abbreviations: N: Not affected by SUZ12 (left of hyphen) or by IFNγ (right of hyphen); I: Interferon-γ; Z: SUZ12; R: Repressed; S: Stimulated; G: Gene.

*Gene classes:*

1. *N-ISG,* ***N****ot affected by SUZ12, but an* ***I****FNγ* ***S****timulated* ***G****ene (****ISG****)*
2. *ZR-ISG, SU****Z****12-****R****epressed* ***ISG***
3. *ZRG-N, SU****Z****12* ***R****epressed* ***G****ene, but* ***N****ot affected by IFNγ*

**Fig P. siSUZ12 induced genes are enriched in CCRI pathway components in multiple cancer cell lines.** GSEA enrichment plots are shown for the 8 cancer cell lines and 3 non-cancer derived lines analyzed here by RNAseq, and for the non-cancer-derived HEK293T/17 cell line RNAseq data obtained from the literature. Significant in GSEA: FDR < 0.05 and NOMpval < 0.01. The top portion of each plot (green line) shows the running Enrichment Score (ES) for the CCRI gene set as the analysis walks down the input ranked gene list. The middle portion (vertical black bars) shows where each member of the CCRI gene set appears in the ranked gene list. The bottom portion (gray) shows the value of the ranking metric, which is the probability of differential expression by siSUZ12 *vs*. control. The three parameters below the graph indicate the Rank of the CCRI gene set on the top 20 reported enriched pathways, the nominal P value (NOM_pval) which estimates the statistical significance of enrichment, and the false discovery rate (FDR). * indicates the 4 cancer cell lines where CCRI was the top or second ranked gene set.

**Fig Q. PRC2 repressed cytokine-cytokine receptor genes.** (A) KEGG pathway mapping of cytokine-cytokine receptor interaction (CCRI) pathway. Of the total of 249 CCRI genes 138 were unaffected (green rectangles) while 111 genes were repressed by PRC2 (Pink rectangles, q > 0.9). The square to the left of each gene indicates the number of cell lines in which the gene was repressed (color key bottom right). (B) Pie graph summarizes the % of PRC2 repressed CCRI genes in > 1 cell lines.

**Fig R. EZH2 inhibitors reduce PRC2 activity in multiple cancer cell lines. (A)** Western blot analyses of the indicated factors, H3K27me3 and H3 were performed using lysates from HeLa, MCF7, A549 and AsPC1 cells treated with DMSO, GSK343 or UNC1999 ± IFNγ for 6 hrs, as indicated in blue above the blots. **(B)** Graphs show the quantification of Western blots in (A) using Actin as loading control. Changes in protein levels were expressed as fold above the control (DMSO/no IFNγ). Values are mean (n=3) +/- SD. *: Significant (p<0.05) induction by IFNγ compared to the matching no IFNγ group. #: Significant (p<0.05) reduction by drug compared to DMSO control.

**Fig S. EZH2 inhibitors augment IFNγ responsiveness of ZR-ISGs in multiple cancer cell lines.** Real-time PCR analysis of ISGs identified by RNAseq as SUZ12 repressed (ZR-ISGs), unaffected by SUZ12 (N-ISG) and non-ISGs in MCF7, A549, HeLa and AsPC1 cells treated with DMSO, GSK343, or UNC1999 ± IFNγ for 6 hrs, as indicated in blue below the graphs. Gene expression was normalized to Tubulin. Values are mean (n=3) +/- SD. * p<0.05 and ** p<0.01 (Student’s t-test) indicate significant increase of IFNγ responsiveness by the drug compared to DMSO+IFNγ control. # p<0.05 indicate significant drug-induced increase in basal expression compared to DMSO control.

*Gene classes:*

1. *ZR-ISG, SU****Z****12-****R****epressed* ***ISG***
2. *N-ISG,* ***N****ot affected by SUZ12, but an* ***I****FNγ* ***S****timulated* ***G****ene (****ISG****)*
3. *N-N, Not affected by SUZ12 and/or IFNγ*

**Fig T: SUZ12 depletion boosts multiple immune pathways.** A549 cells treated with siCtrl or two independent siRNAs for SUZ12 (siSUZ12, siSUZ12_B) were stimulated with the indicated concentrations of TNFα, IFNγ, IL1β, or LPS for 24 h and ELISA was performed for secreted IL6 **(A)**, IL8 **(B)** and CXCL10 **(C)**. Asterisks indicate significant effects (P < 0.05; n = 3; ANOVA followed by Fisher test) according to the indicated comparisons.
